# Influence of Basis Set Composition on Metabolite Quantification of ^1^H‐MRS at 3 T: Combining In Silico, In Vivo and In Vitro Evidence

**DOI:** 10.1002/nbm.70230

**Published:** 2026-02-11

**Authors:** Polina Emeliyanova, Laura M. Parkes, Caroline Lea‐Carnall, Stephen R. Williams

**Affiliations:** ^1^ School of Health Sciences, Faculty of Biology, Medicine and Health The University of Manchester Manchester UK; ^2^ Geoffrey Jefferson Brain Research Centre Manchester Academic Health Science Centre Manchester UK

**Keywords:** basis set models, bias–variance trade‐off, glutamate, in vivo data, linear combination of basis spectra, phantom, synthetic data

## Abstract

Magnetic resonance spectra are quantified by model fitting in the time or frequency domain to a basis set consisting of metabolites present in the measured sample. Despite some work on basis set composition, it remains unclear which metabolite components should be included in the basis set models and how these impact the quantification of key metabolites such as glutamate (Glu). This lack of consensus contributes to reproducibility issues across research groups. Here, we use synthetic, human brain and phantom data to assess how basis set choice impacts quantification, focusing on the bias–variance trade‐off under different SNR conditions. Simulated 1D spectra mimicking in vivo human brain data at 3 T were used to identify basis models that minimise bias and variance for our four key metabolites of interest: Glu, creatine (tCr), choline‐containing compounds (tCho) and N‐acetylaspartate (tNAA) under increasing noise levels. This informed analyses of human brain and phantom data collected at 3 T over 81 and 120 min, respectively, using PRESS. We find that basis set composition significantly affected Glu, tCr, tCho and tNAA concentrations. Specifically, the inclusion of γ‐aminobutyric acid, glutathione, N‐acetylaspartylglutamate and glucose improved Glu quantification, achieving bias and variance below 10%. Including partner metabolites for tCr and tNAA (phosphocreatine and N‐acetylaspartylglutamate) offered no significant benefit. In contrast, tCho quantification remained inconsistent likely due to spectral overlap. We show that minimal basis set models provide accurate quantification while reducing variance. However, the number of metabolites accurately modelled depends on data quality and SNR. High‐SNR spectra enable the inclusion of additional metabolites, while low‐SNR data risk overfitting. Differences in metabolite concentrations reported in the literature may partly reflect variations in prior knowledge models, emphasising the need for clear descriptions of analysis methods in MRS research.

## Graphical abstract

We use synthetic (Aim 1), human brain (Aim 2) and phantom (Aim 3) data to assess how basis set choice affects Glu, tCr, tNAA and tCho quantification, focusing on the bias–variance trade‐off under varying SNR conditions. Including GABA, GSH, NAAG and glucose improved Glu estimates, reducing bias and variance below 10%. Partner metabolites for tCr and tNAA showed no added benefit, while tCho quantification remained inconsistent. Across all methods, minimal basis sets enabled accurate quantification with reduced variance.

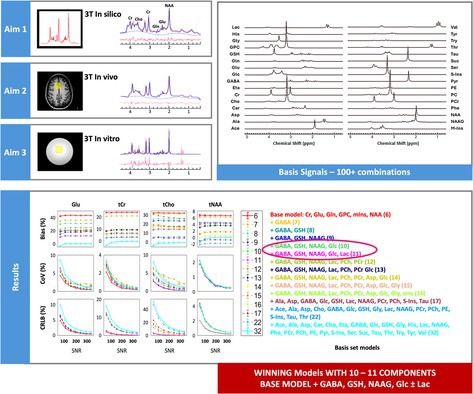

**Abbreviations**
ACC: anterior cingulate cortex
Cr: creatine
CRLB: Cramér–Rao lower bound for uncertainty
CSF: cerebrospinal fluid
GABA: γ‐aminobutyric acid
Glc: glucose
Gln: glutamine
Glu: glutamate
GM: grey matter
GPC: glycerophosphorylcholine
GSH: glutathione
GT: ground truth
HLSVD: Hankel Lanczos singular value decomposition
IR‐STEAM: Inversion recovery STimulated Echo Acquisition Mode
Lac: lactate
m‐Ins: *myo*‐inositol
MM: macromolecules
NAA: N‐acetylaspartate
NAAG: N‐acetylaspartylglutamate
NSA: number of spectral averages
PCh: phosphorylcholine
PCr: phosphocreatine
PRESS: Point RESolved Spectroscopy
QA: quality assessment
RMSE: root‐mean‐squared error
SD: standard deviation
SNR: signal‐to‐noise ratio
tCho: total choline (glycerophosphorylcholine + phosphorylcholine)
tCr: total creatine (creatine + phosphocreatine)
TI: inversion time
tNAA: total N‐acetylaspartate (N‐acetylaspartate + N‐acetylaspartylglutamate)
WM: white matter

## 1 Introduction

Proton magnetic resonance spectroscopy (^1^H‐MRS or MRS) is a powerful non‐invasive technique for investigating brain metabolism and function in both health and disease [1, 2, 3]. The MRS signal acquired from tissue within the volume of interest can be used to calculate concentrations of certain metabolites present in the brain [4, 5], which may be used as biomarkers of neuronal integrity, energy metabolism and pathology (see for review Rae [2]). Linear combination model fitting (LCM) is the most widely used and expert‐recommended approach for quantifying metabolite concentrations from in vivo spectra [4, 6]. However, accurate and precise quantification remains challenging due to several factors, including extensive spectral overlap among metabolites with nearby resonances (e.g., γ‐aminobutyric acid [GABA] and glutamate [Glu]), low signal‐to‐noise ratio (SNR) and incomplete prior knowledge regarding background macromolecular (MM) signals and line shapes [4, 7]. The clinical utility and reproducibility of MRS are further limited by the lack of consensus on optimal modelling strategies, particularly the choice of prior knowledge for basis set compositions and the handling of background signals.

In LCM, MR spectra are quantified by model fitting where a parameterised model function is optimised to explain the data via a nonlinear least‐squares minimization algorithm. Metabolite parameters are estimated in the time or frequency domain using a known model of the metabolites present in the measured sample [4, 8, 9]. Various LCM quantification algorithms have been developed to obtain estimates of metabolite concentrations from MRS, including frequency‐domain methods such as LCModel [10] and OSPREY [11], as well as time‐domain approaches such as TARQUIN [12], QUEST [13] and AQSES [14], the latter two as part of the jMRUI platform [15]. Numerous other algorithms have also been proposed (see for review Graveron‐Demilly [16]). The choice of algorithm typically depends on the specific application, data type and user preference. As such, all analyses in this work were conducted using the QUEST‐based algorithm, ranked among the best in the recent ISMRM'18 MRS Fitting Challenge [17].

The prior knowledge used in these models is represented by a basis set, which contains the individual spectra (basis functions) of metabolites thought to be present in the tissue. Basis sets may be derived experimentally or generated computationally, with the latter being more common [18]. Simulated basis sets are created using density matrix formalism with known physical and chemical characteristics of each metabolite and ensuring compatibility with the pulse sequence parameters used in data acquisition [13, 19].

Since metabolite detectability varies with magnetic field strength, it can substantially influence the choice of basis set composition. Limited spectral resolution at clinical field strengths (1.5–3 T) leads to overlap between metabolite resonances, preventing some metabolites from being estimated independently. The resulting spectrum often includes significant contributions from MM components and a pronounced baseline, both of which can bias metabolite quantification, particularly for metabolites with coupled resonances, such as Glu [7, 20]. These challenges are further exacerbated at low SNR, where inaccuracies in baseline estimation make reliable quantification even more difficult. Under such conditions, it is not clear if including low concentration or weakly represented metabolites (e.g., glutathione [GSH] and glycine [Gly]) would be beneficial, as they are often below the detection threshold without optimised acquisition methods [21, 22, 23, 24]. Consequently, an excessively large basis set may lead to overfitting, with noise or lineshape variations being misattributed to metabolite signals, whereas an overly limited basis set can result in unmodelled or poorly modelled spectral peaks. Therefore, the number of metabolites that can be reliably modelled depends strongly on data quality: High‐SNR, well‐resolved spectra can accommodate more metabolites that would otherwise cause overfitting in lower SNR data. Thus, fitting MRS data in the presence of noise always involves a bias–variance trade‐off. Minimal basis sets, particularly under low‐SNR conditions, increase bias in estimates due to modelling errors caused by the incompleteness of the model. However, this increase in bias is smaller than the corresponding increase in parameter variance. In contrast, larger basis sets include more unknown parameters, which lead to greater variance, resulting in less precise estimates and higher fitting uncertainties [16, 25].

Current literature shows substantial variability in the number of basis set functions used for unedited LCM fitting, typically ranging from approximately 6 to 22 components. Surprisingly, this variation appears largely independent of magnetic field strength. For example, some studies conducted at 1.5–2 T have used a minimum of 15 metabolite components [26], increasing to 17 components [10] and up to 22 components [27]. At 3 T, the most common field strength for in vivo MRS, there is similarly wide variation in basis set size. Some research groups employ relatively small models with fewer than 15 components, such as six or nine [28, 29] or 11 components [24], whereas others use larger basis sets of 15–19 components, which seem to be a more common approach. Examples include Demler et al.'s study using 15–19 components basis models [30]; others include 16 components [31, 32], 17 components [11] and 18 basis components [33]. At 4 T, reported basis set sizes also vary, from as few as 13 components [25] to as many as 20 [34]. Lastly, studies at 7 T similarly demonstrate a broad range, with as few as eight metabolites included [35] and as many as 20 [36], with intermediate examples of 11 [18] and 16 basis components [32].

This lack of standardisation emphasises the need for systematic evaluation of how basis set size influences quantification performance across different field strengths. Despite some work on basis set choice [27, 30, 37, 38, 39, 40], there is no consensus or clear recommendations on which spectra should be included in the basis set models and how different compositions could impact the quantification of key metabolites such as Glu. This lack of consensus may contribute to reproducibility issues across research groups.

Previous works have addressed some aspects of this problem but in more limited contexts. For example, Hofmann et al. [27] examined whether including related metabolite pairs (e.g., N‐acetylaspartate [NAA] and N‐acetylaspartylglutamate [NAAG], creatine [Cr] and phosphocreatine [PCr]) or low‐concentration metabolites (e.g., GSH) improved model fits at 1.5 T, concluding that all metabolites with concentration estimates exceeding their standard error of the mean should be included. More recently, Demler et al. [30] showed that estimated Glu levels in vivo at 3 T vary substantially depending on basis set composition. The most conceptually similar work is the proof‐of‐concept study by Davies‐Jenkins et al. [40], who used a data‐driven approach to optimise basis set selection with Akaike (AIC) and Bayesian (BIC) information criterion scores, demonstrating that all composed basis sets correctly identified high‐concentration metabolites and produced reasonable fits of the spectra.

To the best of our knowledge, no systematic study has investigated how the choice of basis set components affects the model fit and metabolite concentration estimates combining synthetic, human brain and a brain‐mimicking phantom data. To address this gap, we designed a multimodal study combining these three complementary approaches at 3 T. Synthetic data, with an absolute ground truth (GT), allow for the evaluation of accuracy and precision across basis set models under varying levels of noise, helping to identify optimal basis sets. Phantom data, which provide a controlled environment with known metabolite compositions and GT, enable validation of these models in a realistic but predefined metabolite solution. Lastly, human brain data provide biological relevance, allowing us to test how the identified optimal models perform under real in vivo conditions, where variability and the absence of a GT present additional challenges. To address clinical utility, we further tested basis set models under realistic scanning durations to introduce different levels of noise in both human brain and phantom data. While several criteria for basis set model selection exist, including SNR, metabolite linewidth (LW), bias, coefficient of variation (CoV), correlation coefficients, Cramér–Rao lower bounds (CRLB) and more recently, information criteria such as AIC and BIC [27, 30, 37, 38, 39, 40], we chose to evaluate model performance using bias (accuracy), variance (precision) and CRLB (reliability). These metrics provide a direct and quantitative assessment of the bias–variance trade‐off for individual metabolites [16, 25], offering a more interpretable and robust framework for assessing basis set model performance under varying noise levels and for defining optimal basis set design.

The overarching aim of this study is to systematically test the influence of choice of basis set components on the quantification of four key metabolites of interest: Glu, total (t) choline (tCho), tCr and tNAA. We hypothesise that basis sets with a greater number of metabolite components will perform better at higher SNR, yielding more accurate but less precise metabolite estimates. In contrast, basis sets with fewer metabolite components will perform better at lower SNR, producing more precise but less accurate estimates. To test this hypothesis, we evaluate which basis set compositions enable the most accurate and precise metabolite quantification using the following data:

1. Synthetic data under conditions of
  - increasing noise and
  - increasing noise + background and macromolecular signals.
2. Following from this, validate the results across various scanning durations to introduce different levels of noise in
  iii In vivo human brain data
  iv Phantom data.

## 2 Materials and Methods

### 2.1 Synthetic Data

#### 2.1.1 Basis Sets

**FIGURE 1:**
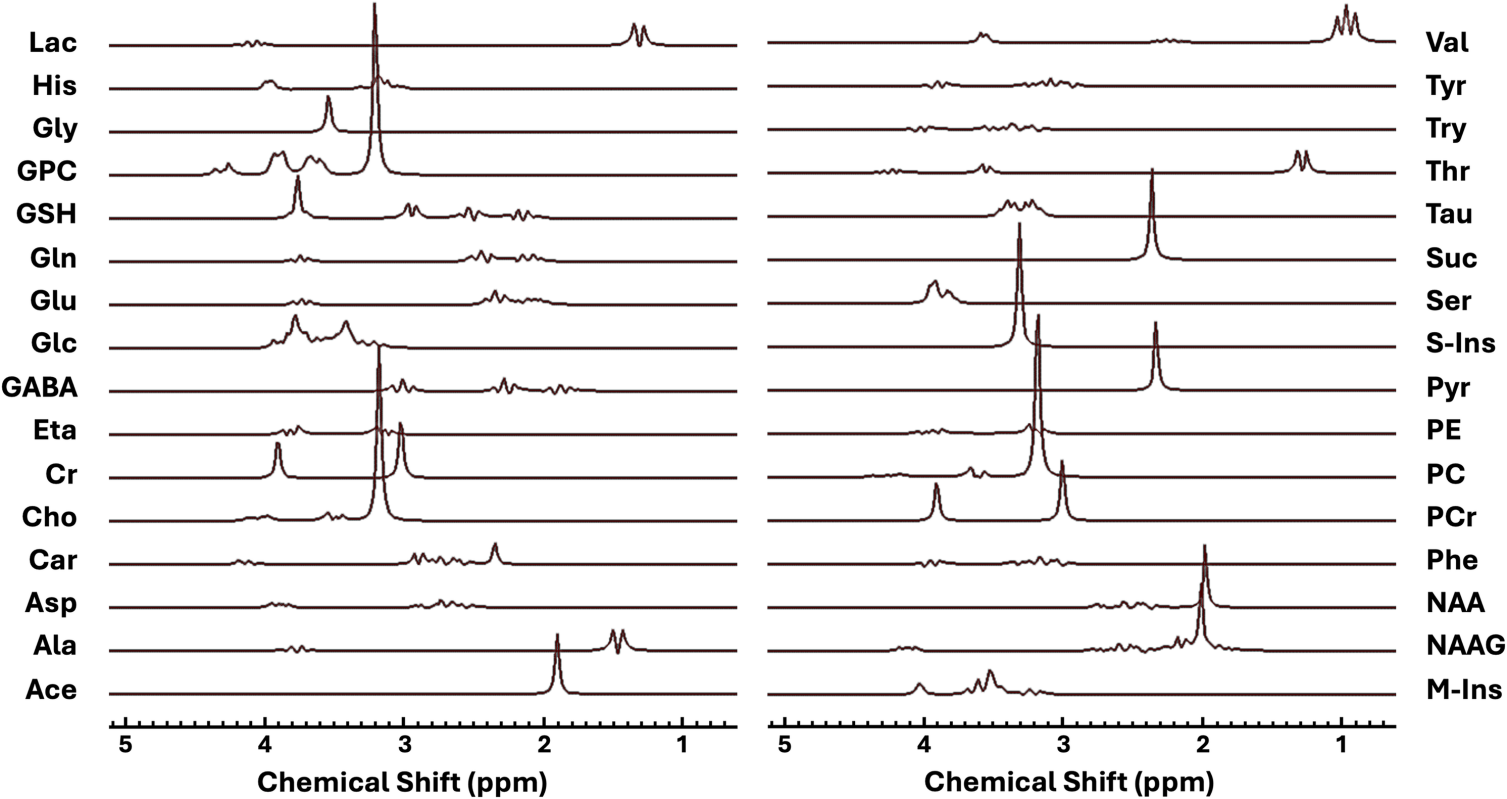
Basis spectra. NMR‐scopeB simulated spectra of 32 metabolites using Philips PRESS sequence at 3 T with TE = 35 ms. NAA = N‐acetylaspartate, NAAG = N‐acetylaspartylglutamate, GABA = γ‐aminobutyric acid, Cho = choline, Cr = creatine, PCr = phosphocreatine, Glu = glutamate, m‐Ins = myo‐inositol, Lac = lactate, Ala = alanine, Ace = acetate, Asp = aspartate, Car = carnosine, Eta = Ethanolamine, Glc = glucose, Gln = glutamine, Gly = glycine, GPC = glycerophosphorylcholine, GSH = glutathione, His = histidine, PC = phosphorylcholine, PE = phosphorylethanolamine, Phe = phenylalanine, Pyr = pyruvate, Ser = serine, Suc = succinate, s‐Ins = scyllo‐inositol, Tau = taurine, Thr = threonine, Try = tryptophan, Tyr = tyrosine, Val = valine.

The theoretical time‐domain model signals of 32 metabolites (thought to be present in the human brain) [2, 41, 42] were quantum‐mechanically simulated using NMR Spectra Calculation using OPErators—Brno (NMR‐scopeB) routines [15, 43, 44] with Philips radiofrequency (RF) pulse shapes for Point RESolved Spectroscopy (PRESS) sequence at 3 T [45] (Figure 1). The simulated parameters matched our in vivo and phantom protocols: TE = 35 ms (TE_1_/TE_2_ = 16/19 ms), bandwidth of 2 kHz and 1024 data samples. For the in vivo analysis, Lorentzian line broadening of 4 Hz was applied to the generated signals to approach (but be less than) the metabolite signal damping in vivo [26], whereas for phantom analysis, line broadening of 1 Hz was applied to the signals. The spin Hamiltonian parameters, including J‐coupling constants, number of spins and chemical shifts, were obtained from previously published values in Govind et al. [46], Govindaraju et al. [41] and Kaiser et al. [23]. Carnosine parameters were obtained from Friedrich and Wasylishen [47]. We generated an in vivo noise‐free composite MM spectrum, which was incorporated into the basis sets alongside the metabolite signals and fitted to the synthetic and acquired human brain spectra. Acquisition and processing of the MM signal are detailed below. This allowed us to address MM contributions present at 3 T, whereas baseline components were handled using QUEST‐Subtract in jMRUI v7 [13].

#### 2.1.2 Synthetic Spectra

First, we generated a synthetic brain spectrum, consisting of all 32 basis set metabolites, assumed to be present in the human brain [2, 41, 42], in which each component was multiplied by a constant, so they were present in the final spectrum at ratios corresponding to the expected concentration ratios in vivo. Each simulated signal was weighted according to the in vivo concentrations of the normal healthy brain in mM units, resulting in NAA—10.3, NAAG—2.7, Cr—4.5, PCr—3.5, choline (Cho)—0.1, phosphorylcholine (PCh/PC)—0.6, GPC—1, *myo*‐inositol (m‐Ins)—8, Glu—12, glutamine (Gln)—4, lactate (Lac)—0.5, threonine (Thr)—0.3, GABA—1, GSH—3, aspartate (Asp)—2, glucose (Glc)—1, Gly—1, acetate (Ace)—0.8, alanine (Ala)—0.5, ethanolamine (Eta)—1.4, Histidine (His)—0.1, Carnosine (Carn)—0.7, *scyllo*‐inositol (s‐Ins)—0.6, phenylalanine (Phe)—0.2, phosphorylethanolamine (PE)—1, pyruvate (Pyr)—0.2, serine (Ser)—1, succinate (Suc)—0.5, taurine (Tau)—1.5, tryptophan (Try)—0.03, tyrosine (Tyr)—0.05 and valine (Val)—0.1 [2, 41, 42, 48, 49]. The resulting spectrum was Lorentzian line broadened by 5 Hz to approximate the in vivo signal at 3 T.

**FIGURE 2:**
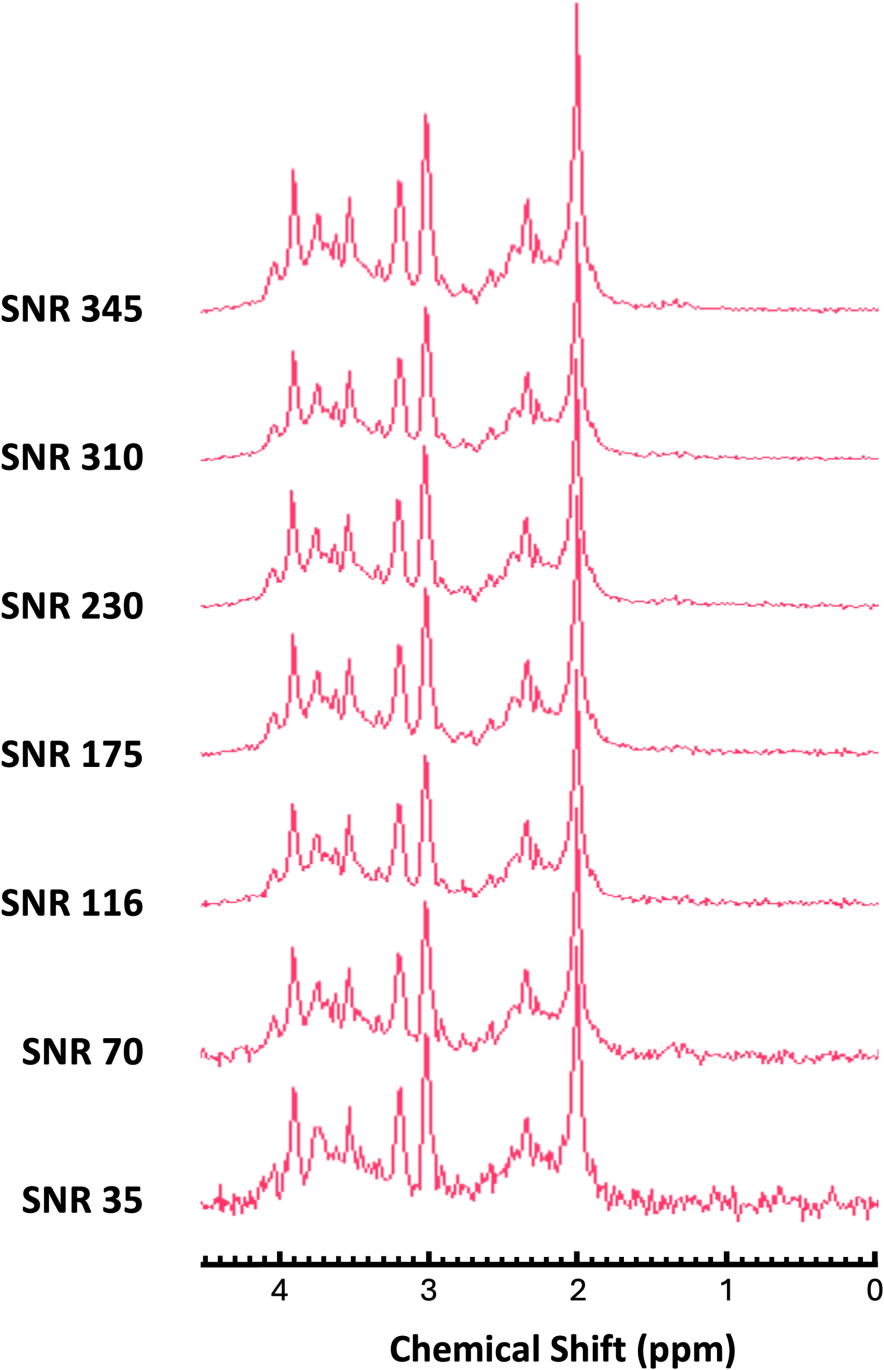
Synthetic human brain‐like spectra. Synthetic data with seven level of noise (SNR 35, 70, 116, 175, 230, 310 and 345) calculated as NAA amplitude/SD noise in the frequency domain.

Second, to explore the effect of choice of basis set components on fitting and quantification in the presence of noise, we generated 100 Monte Carlo simulations of Gaussian‐distributed white‐noise realisations, and these were added to the synthetic noise‐free data. The added noise mimicked SNR levels observed in in vivo acquisitions and was calculated as NAA amplitude/standard deviation (SD) of noise in the frequency domain. We selected a total of seven noise levels, of which five are based on PRESS SNR values of 116, 175, 230, 310 and 345, corresponding to scan times ranging from approximately 2 min (low SNR) to 20 min (high SNR) for our in vivo acquisitions from a 3 × 3 × 3 cm^3^ volume at 3 T (Figure 2). Additionally, we included SNR values of 35 and 70, which are representative of typical ^1^H‐MRS acquisitions from a 2 × 2 × 2 cm^3^ volume at 3 T (~2 and 8 min, respectively). We chose four metabolites of interest: Glu, tCr, tCho and tNAA since they are among the most abundant neurochemicals and are involved in the main metabolic and neurotransmission‐related processes in the brain [2, 42]. Total tCr and tNAA are the most commonly used references, and we expect their concentrations to remain reasonably stable across all basis set compositions. We first chose to assess these signals in the presence of increasing noise only, eliminating possible confounds introduced by the background and MM contributions.

Finally, we added a measured baseline and macromolecular background signal (BGMM) to the noisy synthetic data to explore the effect of BGMM on fitting and quantification of these metabolites and to establish an optimised background handling approach for human brain data analysis using QUEST‐Subtract.

#### 2.1.3 Optimal Basis Sets Selection

The synthetic data approach allowed us to systematically assess the effect of the composition and number of metabolite components in basis set models on the estimated concentrations of Glu, tCr, tCho and tNAA in the presence of increasing noise. The base model was assumed to consist of six prominent signals: Cr, Glu, NAA, GPC, Gln and m‐Ins and was included in all comparisons. Sequentially, 1 to 10 additional metabolite components (with concentrations mostly ≥ 1 mM: NAAG, PCr, PCh, GABA, Lac, GSH, Asp, Glc, s‐Ins and Gly) were added to the base model, forming models with up to 16 components. This stepwise approach enabled us to identify basis sets that minimise bias and variance. In models where only Cr, NAA and GPC are included, tCr, tNAA and tCho are simply the individual metabolites; otherwise, tCr = Cr + PCr, tNAA = NAA + NAAG and tCho = GPC + PCh or tCho = GPC + PCh + Cho.

Each subsequent fitting informed which additional components improved accuracy and precision, guiding their inclusion in the next model. We evaluated models sequentially, with 7, 8, 9, 10, 11, 12, 13, 14, 15 and 16 components, varying model compositions, except six base signals that were always present in the fitting (see Figure S1).

After establishing basis sets with lower bias and variance using up to 16 most prominent signals in the brain, we then explored commonly used basis sets in the literature with components greater than 16 (typically adding metabolites < 1 mM) to evaluate the impact of additional components on accuracy and precision of the estimates for Glu, tCr, tCho and tNAA (Table 1). The choice of the components in the larger basis set models was informed by the previous literature [10, 11, 12, 18, 27, 33, 50]. Among these, the standard LCModel‐recommended basis set emerged as one of the most frequently used.

**TABLE 1:** Optimal basis set models.

No. of metabolites	Metabolites
6^a^	Cr, Glu, Gln, GPC, m‐Ins, NAA
7	Base model + GABA
8	Base model + GABA, GSH
9^a^	Base model + GABA, NAAG, GSH
10^a^	Base model + GABA, NAAG, GSH, Glc
11^a^	Base model + GABA, NAAG, GSH, Glc, Lac
12^a^	Base model + GABA, NAAG, GSH, Lac, PCh, PCr
13	Base model + GABA, NAAG, GSH, Lac, PCh, PCr, Glc
14^a^	Base model + GABA, NAAG, GSH, Lac, PCh, PCr, Asp, Glc
15	Base model + GABA, NAAG, GSH, Lac, PCh, PCr, Asp, Glc, Gly
16	Base model + GABA, NAAG, GSH, Lac, PCh, PCr, Asp, Glc, Gly, s‐Ins
17^a^	Base model + Ala, Asp, GABA, Glc, GSH, Lac, NAAG, PCr, PCh, s‐Ins, Tau
22^a^	Base model + Ace, Ala, Asp, Cho, GABA, Glc, GSH, Gly, Lac, NAAG, PCr, PCh, PE, s‐Ins, Tau, Thr
32^a^	Base model + Ace, Ala, Asp, Car, Cho, Eta, GABA, Glc, GSH, Gly, His, Lac, NAAG, Phe, PCr, PCh, PE, Pyr, s‐Ins, Ser, Suc, Tau, Thr, Try, Tyr, Val

*Note:* Optimal basis sets were selected in each sequential model comparison for 7, 8, 9, 10, 11, 12, 13, 14, 15 and 16 components using synthetic data. The analysis included 6–16 component models as well as models with 17, 22 and 32 components sourced from the literature.

^a^ Nine optimal basis set models were carried forward for follow‐up analyses in BGMM synthetic, human brain and phantom data. Base model = ‘Cr, Glu, Gln, GPC, m‐Ins, NAA’.

The model evaluation guided the selection of basis sets for follow‐up analyses. The final outcome identified five optimal basis sets (< 16 components) that showed lower bias and variance, along with two commonly used basis sets from the literature (see Table 1). A basis set containing six base signals and a full 32 component model were also evaluated. The latter served as a ‘sanity check’ but was not considered in the comparisons. These nine basis sets (Table 1) were subsequently tested on synthetic data with added BGMM patterns, as well as in vivo and phantom data with varying levels of noise. The goodness of model fits was evaluated using bias, CoV, root‐mean‐squared error (RMSE) and CRLB for metabolite estimate uncertainty.

### 2.2 Human Brain Data Acquisition

**FIGURE 3:**
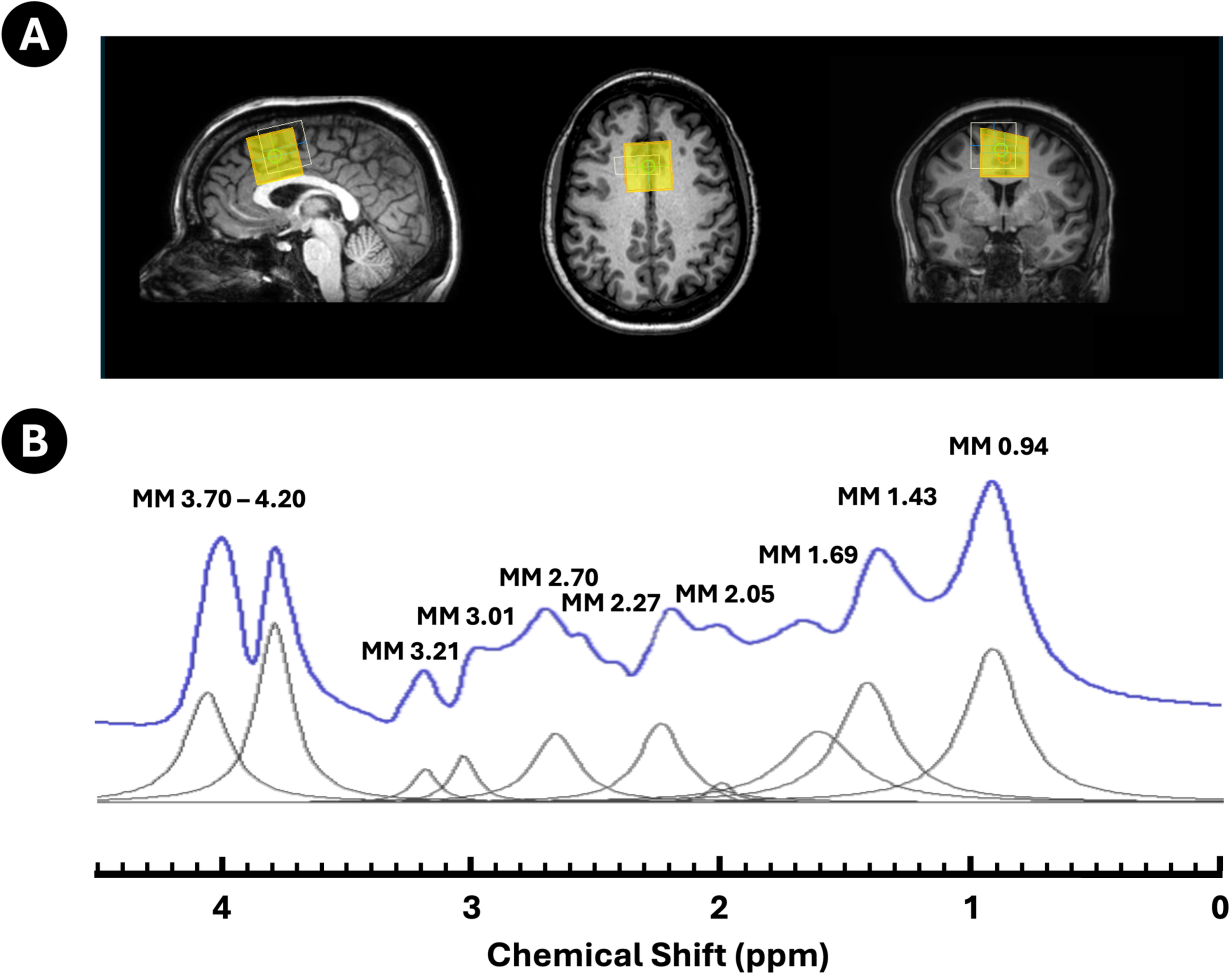
Human brain data. (A) Example of a 27 mL voxel placement on a structural scan of a single subject, showing sagittal, axial and coronal slices centred over the anterior cingulate cortex. The voxel placement is shown in yellow with chemical shift displacement in white and the shim box in green. (B) The noise‐free MM spectrum and MM model signals employed in the fitting. The residual metabolites were subtracted prior spectrum parameterization. The 10 macromolecule resonances are depicted in grey, and 10‐Hz line broadening was used for visualisation purposes.

MR data were collected using a 3 T Phillips Achieva TX scanner (Philips Healthcare, The Netherlands) with a 32‐channel head coil for signal acquisition. Standard second order pencil beam shimming ensured good water suppression and optimal signal resolution. The localization of ^1^H‐MRS voxel was based on 3D T_1_‐weighted MPRAGE (Magnetization‐Prepared Rapid Gradient‐Echo) images (TR/TE/TI = 12/5.7/900 ms). We acquired 81 min of Philips PRESS [45] data as well as a measured MM spectrum from a 3 × 3 × 3 cm^3^/27 mL voxel positioned in the anterior cingulate cortex (ACC), encompassing both hemispheres, in a single subject (Figure 3A) (TE/TR = 35/1500 ms, TE_1_/TE_2_ = 16/19 ms, 1024 spectral points, BW = 2 kHz, 270 dynamics with NSA = 12 per dynamic). An unsuppressed interleaved water reference scan (NSA = 12) was collected to be used as an internal concentration reference. The metabolite‐nulled acquisition (TE/TR/TI = 35/1500/600 ms) was collected over 10 min using the inversion recovery (IR) PRESS, resulting in 23 dynamics with NSA = 17 per dynamic [51]. Informed consent was obtained from the subject prior to participation in the study.

### 2.3 Phantom Data Acquisition

The brain‐mimicking phantom was designed to contain the brain's major metabolites according to Govindaraju et al. [41] and Rae [2]. To increase the SNR, we added 32 metabolites at five times physiological concentrations with values adapted from the literature [41] (see Table S1). A ready‐made gold standard phantom (GSP, London, UK), containing an aqueous solution with a selection of seven primary metabolites, was used as a base to which additional metabolites were added (Table S1). The additional metabolites were prepared in de‐ionised water and, when combined with the GSP phantom, the final solution pH was adjusted to pH = 7.3. Most chemicals were acquired from Glentham Life Sciences (Corsham, Wiltshire, UK), except GPC, PCh and s‐Ins, which were sourced from BOC Sciences (Shirley, New York, USA). The phantom temperature was maintained at 20°C for data acquisition. MRS data were collected from a 3 × 3 × 3 cm^3^ voxel positioned at the centre of the spherical phantom for 120 min using the in vivo PRESS protocol. TR was relatively long and set to TR = 8 s to minimise T_1_ relaxation effects, resulting in 180 dynamics with NSA = 5 per dynamic. An unsuppressed interleaved water reference scan (NSA = 5) was collected to quantify metabolite concentration values.

To correct for T_1_ and T_2_ effects in the phantom, we collected IR and TE series in a separate session. To measure T_1_, the IR series were acquired with IR‐PRESS sequence (4096 points/2‐kHz bandwidth/TR 10 s) without water suppression using fixed TE = 35 ms and variable inversion time (TI) with 9 values from 100 to 7000 ms. To measure T_2_, we used a fixed TR = 4 s and variable TE with 7 values ranging from 35 to 1500 ms, with separate unsuppressed water scans collected at each TE.

### 2.4 Analysis

#### 2.4.1 Synthetic Spectra Analysis

All synthetic spectra were analysed in jMRUI v7. First, each set of 100 in silico Monte Carlo simulations (seven datasets, one for each noise level) was fitted and quantified in QUEST using simulated basis set models. This process generated one dataset per model fitting for each of the seven SNR levels, resulting in over 100 basis set model comparisons (see Figure S1 for model selection). The GT was determined by the weightings of the basis set signals in the synthetic spectrum. From this step, we identified nine optimal basis sets (Table 1) for follow‐up analyses.

Next, BGMM synthetic data were analysed using the nine optimal basis sets identified during model selection (Table 1) to evaluate the effect of BGMM on quantification and to establish a background handling approach for subsequent in vivo data analysis. To achieve this, we employed the QUEST‐Subtract algorithm, a method specifically designed to handle baseline and MM signals [17, 52]. This algorithm estimates metabolite components after truncation of the early time‐domain points to separate the rapidly decaying baseline components only present at the beginning of the FID [25]. This approach decorrelates metabolite and ‘nuisance’ background signals, improving quantification estimates [16, 52]. The isolated baseline time‐domain signal can then be subtracted (‘pseudo‐truncated’) from the original FID, thus considerably flattening the baseline. Combining this with simulated or measured in vivo MM components can further improve quantification. The number of truncated points in QUEST‐Subtract is defined interactively, and isolating too few or too many may result in incorrect quantification with overestimating or underestimating the metabolite concentrations [13]. To explore the impact of truncation on baseline handling and metabolite quantification, we tested five different truncation strategies: truncating 10 points (5 ms), 15 points (7.5 ms), 20 points (10 ms), 30 points (15 ms) and 40 points (20 ms) in the time domain, allowing us to determine which approach would minimise bias and variance of the estimated parameters.

#### 2.4.2 Human Brain Data Analysis

Human brain data were fitted and quantified with a standard processing pipeline in jMRUI QUEST [13], including frequency alignment to NAA singlet at 2.02 ppm and residual water removal with the Hankel Lanczos singular value decomposition (HLSVD) routine [53]. Spectra were quantified for a range of durations: every 1.8 min up to 27 min and then every 9 min up to 81 min of total duration. This approach enabled us to vary SNR levels and evaluate the optimal basis set choice under increasing noise levels. For each duration, data were bootstrapped by resampling with replacement 100 times to allow calculation of the mean and SD for each metabolite for each duration [54]. Each sampled duration was then fitted with nine optimal basis sets defined in the sequential synthetic spectra analysis (Table 1). The zero‐order phase and beginning time were set to zero, while damping factors, frequencies and amplitudes remained as free parameters in the quantification algorithm. Metabolite linewidths were constrained to be equal within a spectrum on the assumption that the linewidth is largely determined by the field inhomogeneity across the voxel.

The in vivo MM data were processed in jMRUI v7 following the recommendations of Hui et al. [51] and Cudalbu et al. [7]. Residual metabolites were first removed from the measured MM spectrum using a reduced basis set (Cr, NAA, Glu and GPC), resulting in a ‘residual‐metabolite‐free’ MM spectrum. This spectrum was then parameterised and modelled using the HLSVD method to fit Gaussian peaks between 0.5 and 4.2 ppm, excluding components outside of the fitting range. This produced a noise‐free MM spectrum (Figure 3B). The noise‐free composite MM baseline was incorporated into the basis sets as a single component, alongside the metabolite signals, and fitted to the acquired and simulated human brain data. The BGMM signal in the human brain data was addressed with the QUEST‐Subtract option, which models broad components in the spectrum as a sum of fast decaying, exponentially damped sinusoids [13, 52]. Here, we used 10 ‘pseudo‐truncated’ points (= 5 ms), a value determined in the BGMM synthetic data analysis step.

In vivo metabolite concentrations for Glu, tCr, tCho and tNAA were calculated relative to an unsuppressed water reference scan (Equations 1 and 2) following the methods described in Gasparovic et al. [5] and Near et al. [4]. Correction for partial volume effects was achieved by establishing the percentage of each tissue type in the spectroscopic voxel. The T_1_‐MPRAGE image was segmented in MATLAB R2022a (The MathWorks, Natick, Massachusetts, USA) using statistical parametric mapping SPM 8 (http://www.fil.ion.ucl.ac.uk/spm/) to extract % grey matter (GM), % white matter (WM) and % cerebrospinal fluid (CSF) within the voxel. Voxel registration was performed using a custom‐made MATLAB script (provided by Dr. Hamied Haroon from the University of Manchester), which generated a mask for voxel location by combining location information from the Philips SPAR file and the orientation and location information in the T_1_ image. Molar concentration of pure water was scaled according to the tissue water density [55] and by the volume fraction of tissue in the voxel [4]. Human brain data metabolite concentrations (mM) were corrected for % CSF and tissue‐specific water T_1_ and T_2_ relaxation time with values adapted from the literature [56, 57, 58]. Metabolite T_1_ and T_2_ correction was not applied, as it would have a relatively minor differential effect among metabolites at TE/TR = 35/1500 ms [4] and in any case was obviated by our decision to use as GT the full 81 min acquisition. The GT for the major metabolites of interest (tNAA, tCr, tCho and Glu) in the human brain data was derived from fitting the full acquisition duration (81 min) using a basis set model containing 17 molecular components. This basis model was adapted from the LCModel manual (http://s‐provencher.com/lcm‐manual) and is commonly used in the literature (Table 1).
(1)

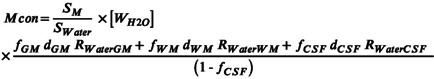

where *S*_*M*_ and *S*_*Water*_ are the fitted signal amplitudes of metabolite and water, *W*_*H2O*_ is the [^1^H] concentration of pure water (111.02 mol/kg), *f* is the volume fraction of *GM*, *WM* and *CSF* in the voxel and *d* is water density in *GM* (0.78), *WM* (0.65) and *CSF* (0.97). *R*_*Water*_ is a relaxation scaling factor for water in different compartments [4, 5]. In QUEST, the fitted signal amplitude *S*_*M*_ already takes into account the number of protons contributing to the metabolite spectrum.

(2) $$R=e^{-\mathrm{TE}/T_{2}}\left[1-e^{-\frac{\mathrm{TR}}{T_{1}}}\right]$$

where *R* is a relaxation scaling factor for water or metabolites, *TE* = echo time, *TR* = repetition time, *T*_2_ and *T*_1_ are the relaxation times for water/metabolites. Water GM: T_1GM_ = 1331 ms / T_2GM_ = 110 ms, WM: T_1WM_ = 832 ms / T_2WM_ = 79.6 ms, CSF: T_1CSF_ = 4163 ms / T_2CSF_ = 2000 ms [56, 57, 58].

#### 2.4.3 Phantom Data Analysis

The analysis for the phantom data was similar to the human brain data. Here, bootstrapped and resampled spectra were also quantified for a range of durations: every 2 min up to 30 min and then every 10 min up to 120 min of total duration, enabling us to achieve variable SNR levels to evaluate the optimal basis set choice under increasing noise levels. Each sampled duration was fitted with the nine optimal basis sets previously identified (Table 1).

Metabolite concentrations (mM) in the phantom were calculated relative to the water unsuppressed scan. The concentrations for Glu, tNAA, tCr and tCho were corrected for T_1_ and T_2_ relaxation of both metabolites and water (Equations 1 and 2). Metabolite and water T_1_ estimates (T_1water_ = 2766 ms, T_1CR_ = 1775 ms, T_1GLU_ = 986 ms, T_1NAA_ = 1159 ms, T_1NAAG_ = 855 ms, T_1GPC_ = 1927 ms) and T_2_ estimates (T_2water_ = 400 ms, T_2CR_ = 805 ms, T_2PCR_ = 740 ms, T_2GLU_ = 473 ms, T_2NAA_ = 788 ms, T_2NAAG_ = 706 ms, T_2GPC_ = 843 ms) were obtained from the nonlinear least‐squares fit (‘lsqcurve’) of the amplitude of the fitted time‐domain signal measured at each TI and TE value, respectively (Figures S3 and S4). Separate basis sets, matching acquisition parameters, were created for this purpose.

The potential degradation in the phantom solution was assessed using an 800 MHz Bruker Ascend spectrometer to help define the GT for the phantom analysis. We note that at the ultra‐high field of 18.8 T, small chemical shift inaccuracies in the simulated basis sets [41] prevented reliable QUEST fitting (e.g., NAA methyl singlet vs. aspartyl doublets, Cr methyl vs. methylene singlets could not be simultaneously aligned). We therefore used AMARES (individual peak models approach) [59] to quantify the first and last individual acquisitions over the 5‐h high‐resolution spectrometer run. The results indicate overall stability of the solution, with reliable quantification obtained for well‐separated, strongly represented peaks (e.g., NAA and Cr), whereas several low‐concentration metabolites were not detected or deviated from their initial values (Tables S1 and S2). To determine whether these deviations reflected phantom degradation, we extracted a difference spectrum between the first and last acquisition (5 h later) over the frequency range of 1.2–4.2 ppm. As demonstrated in Figure S14, the expansion from 1.3 to 2.2 ppm in the upper right shows that there was a small frequency drift across the acquisition time resulting in +ve and −ve difference signals which give the visual appearance of integrating close to zero. These findings indicate that the observed deviations in low‐concentration metabolites are more likely attributable to frequency drift rather than chemical degradation, and that overall concentration changes over 5 h at room temperature were negligible.

Therefore, for all subsequent comparisons, we consider the result of fitting the full acquisition duration (120 min) with a 32‐signal basis set, corrected for water and metabolite T_1_ and T_2_ relaxation times and converted into mM units, as the best approximation of GT. This is preferable to the 800 MHz reference (which suffered alignment/quantification limitations at ultra‐high field) and to the initial phantom concentrations (which could not be confidently corrected for T_1_ and T_2_ effects). Thus, the 120‐min/32‐signal fit reflects the measured state of the phantom at acquisition and provides the most accurate GT reference available for our analyses. The quantifications determined from all nine basis set model fittings (Table 1) were compared to this GT.

For additional details, see Appendix S2, where we report consensus‐recommended parameters for our MRS data acquisition, analysis and QA for both phantom and in vivo data [60].

#### 2.4.4 Assessment Metric

We implement an established assessment metric based on bias and variance to evaluate which basis set component combinations enable more accurate and precise metabolite quantification across all of our approaches [16, 25]. Bias (%) was calculated as the percentage deviation of the mean metabolite amplitude from the GT. CoV (%) was expressed as the percentage ratio of the SD to the mean metabolite amplitude. RMSE (mM) was computed as a combined measure of absolute bias and variance. CRLB (%) was determined as the percentage ratio of CRLB values (derived from the QUEST algorithm) to the mean metabolite amplitude (Equations (3), (5)).

(3) Bias%=m−GTGT×100

(4) CoV%=SDm×100

(5) $$\text{RMSE}\;\left(\mathrm{mM}\right)=\sqrt{{\text{Absolute Bias}}^{2}+{\mathrm{SD}}^{2}}$$

where *m* is a mean signal amplitude or metabolite concentration (mM), GT is the ground truth, SD is standard deviation of the data. Absolute Bias = absolute difference between the mean metabolite amplitude or concentration from the GT; CoV = coefficient of variation, RMSE = root‐mean‐squared error. Bias and CoV are expressed as percentages (%), RMSE in mM units.

## 3 Results

### 3.1 Synthetic Spectra

**FIGURE 4:**
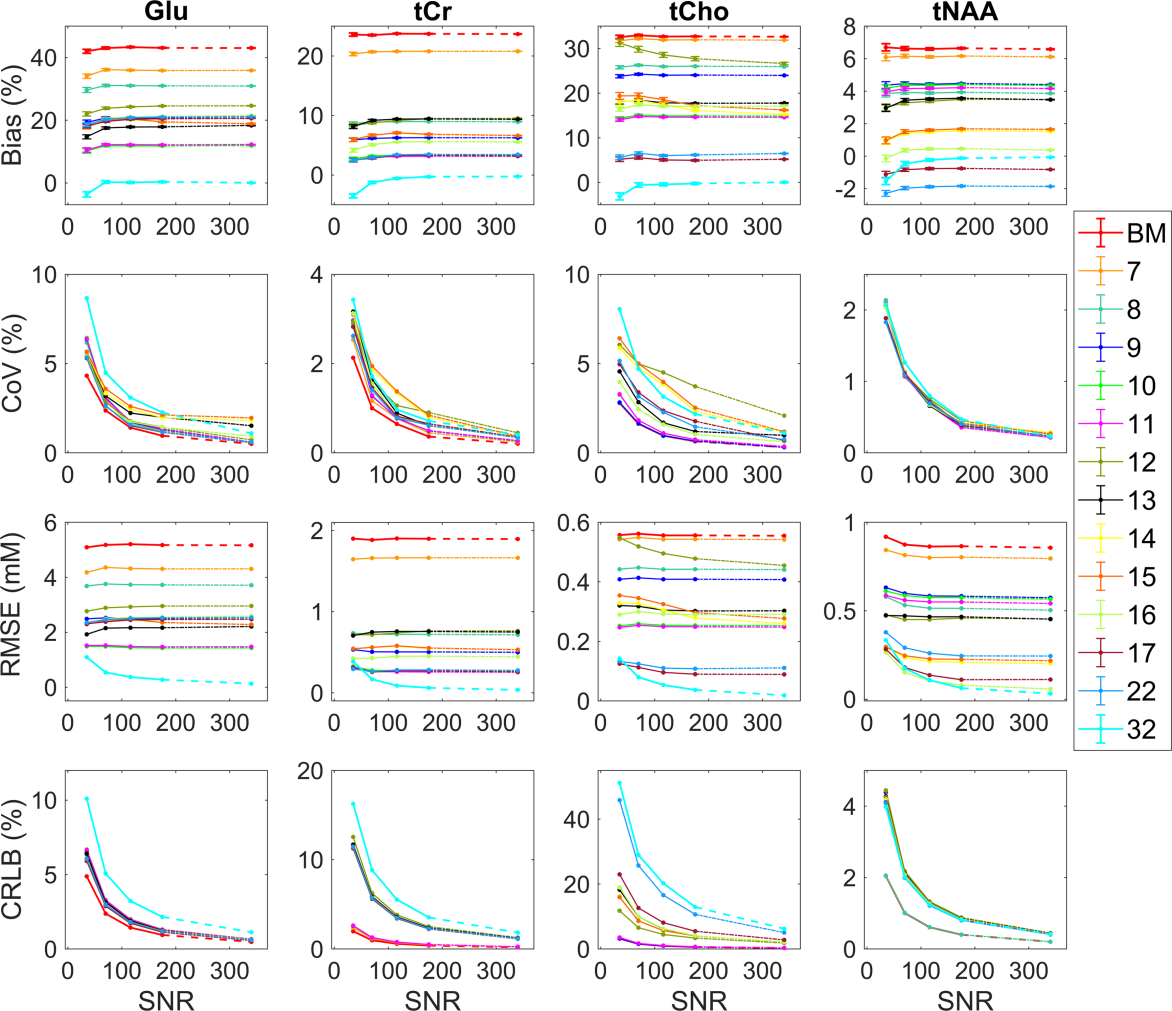
Bias, variance and fitting of synthetic data. Synthetic data results. Glu, tCr, tCho and tNAA quantification with 6–32 basis set components across five SNR levels (35, 70, 116, 175 and 345), matching PRESS in vivo acquisitions. Top panel is showing bias from the ground truth (GT, %) with standard error bars, middle panel is showing variability in the data expressed as coefficient of variation (CoV, %), the third panel is showing root‐mean‐squared error (RMSE, mM) and the bottom panel is showing Cramér–Rao lower bound (CRLB, %) for metabolite estimates uncertainty. The BM = ‘base model’ with six components is shown in solid red line ‘Cr, Glu, Gln, GPC, myo‐Ins, NAA’ and the full 32‐component model in solid cyan line. See Table 1 for the full breakdown of components for each model.

Here, we explore an effect of choice of basis set components on fitting and quantification in the presence of increasing noise and optimise background handling approach for human brain data analysis using QUEST‐Subtract. Only the most relevant results are discussed and are shown in Figure 4 (the full results are available on request).

#### 3.1.1 Optimal Basis Sets—Synthetic Data

First, we assessed fitting outcomes using models with six to 11 basis components for our four key metabolites: Glu, tCr, tCho and tNAA. Addition of the ‘GABA NAAG GSH’ components consistently reduced bias across models with seven to 11 components. The 11‐component model, including ‘Base model + GABA NAAG, GSH, Glc, Lac’, exhibited a bias of ~12% for Glu, compared to ~11% in the 10‐component model (‘Base model + GABA NAAG GSH Glc’), 20% in the 9‐component model (‘Base model + GABA, NAAG, GSH’) and 23%–48% in models with six to eight components (see Figures S5–S9). Bias for tCr (~3.3%) and tNAA (~4.3%) remained stable between the 10‐ and 11‐component models but increased to 6% for tCr in the 9‐component model, while tNAA remained within 4.5%. Models with six to eight components showed a bias increase up to 23% for tCr and up to 7% for tNAA. For tCho, bias was lower in the 11‐component model (~14%) compared to the 10‐component (15%) and 9‐component (24%) models, while models with six to eight components showed increased bias (up to ~30%–34%). We consistently found that inclusion of partner metabolites for tCr, tCho and tNAA did not significantly improve accuracy of these metabolites and increased estimate variability (CoV %), CRLB and RMSE, particularly for tCho. This effect was especially pronounced in lower SNR data (see Appendix S1 for full results).

Following from this, we assessed models with 12–16 components and explored two fully specified basis sets commonly used in the literature (Table 1). Notably, adding more components to the base model increased bias for Glu up to 18%–25% compared to ~11%–12% in models with 10‐ and 11‐components (‘Base model + GABA, NAAG, GSH, Glc ± Lac’). Bias for tCr remained stable at ~3% in larger models with ≥ 17 components, similar to minimal models with 10–11 components, whereas models with 12–16 components showed increased bias of 6–9%. In contrast, bias reduction in tNAA signals (4% to −2%) was observed in basis models with 12–32 components, while bias reduction in tCho (15% to 5%) was noted in basis models with ≥ 17 components. The CRLB and CoV increased in all larger models with lower SNR levels. Inclusion of partner metabolites (PCr, NAAG and PCh) for tCr, tNAA and tCho in the basis sets notably increased bias, variability, RMSE and CRLB for all three metabolites. Figure 4 demonstrates that the CoV is lower in models with a reduced number of components compared to fully specified models. The SDs were approximating CRLB values for all four metabolites of interest in high to medium SNR conditions, whereas low‐SNR datasets yielded less agreement between these values.

Overall, basis models with 10–11 metabolite components (‘Base model + GABA, NAAG, GSH, Glc ± Lac’) largely outperform more specified models, as well as models with fewer than 10 components, for Glu, yielding more accurate results. tNAA estimates demonstrate robustness to the basis set size and SNR, with consistent performance across all metrics (within 10%). tCho yields more accurate but less precise estimates with larger models, whereas tCr generally offers accurate and precise estimates (within 10%) for all models > 7 components. As expected, the model with 32 components achieved the highest accuracy but the lowest precision, particularly at lower SNR. The variability in estimated parameters and CRLBs showed a clear negative relationship with SNR, increasing as noise levels increased. However, no consistent relationship between noise and bias was observed (Figure 4). RMSE tends to decrease with increasing SNR for all metabolites. While all metrics improve with increasing SNR, the benefit diminishes at very high‐SNR levels. Thus, this analysis identified nine optimal basis sets that minimised bias and variance, providing a foundation for subsequent analyses of BGMM synthetic and measured human and phantom data in the follow‐up sections (Table 1 shows final optimal basis sets).

#### 3.1.2 Effect of BGMM on Quantification

Here, we investigate the effect of the BGMM signal on fitting performance in the presence of increasing noise. We evaluated five different truncation strategies (10–40 data points, covering the first 5–20 ms of the acquisition).

**FIGURE 5:**
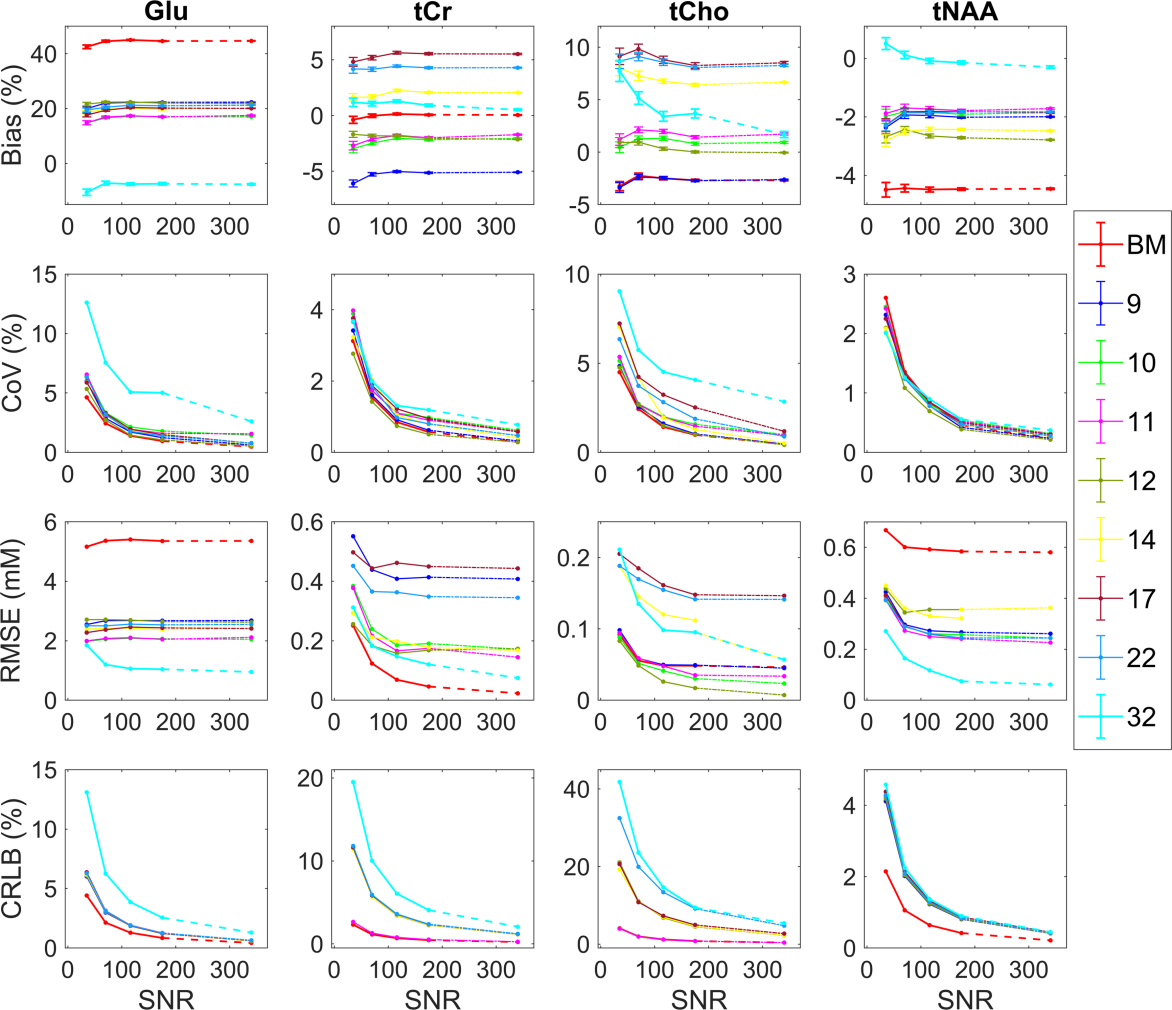
Bias, variance and fitting of synthetic data with BGMM. Results for synthetic BGMM data analysis. Glu, tCr, tCho and tNAA quantification with nine basis sets across five SNR levels (35, 70, 116, 175 and 345), using QUEST‐subtract background handling with 10 truncated points. Top panel is showing bias from the ground truth (GT, %) with standard error bars, middle panel is showing variability in the data expressed as coefficient of variation (CoV, %), the third panel is showing root‐mean‐squared error (RMSE, mM) and the bottom panel is showing Cramér–Rao lower bound (CRLB, %) for metabolite estimates uncertainty. See Table 1 for the full breakdown of basis sets components. BM = base model.

Metabolite quantification was influenced by both the number of truncated points and the basis set size. The results indicate that handling the BGMM signal with 10 truncated points (Figure 5) minimises bias and data variability compared to using a larger number of truncated points (Figures S10–S13).

Increasing truncation from 10 to 40 points systematically increased both bias and variance across metabolites. In low‐SNR datasets, Glu bias with minimal models (6‐ and 9‐component basis sets) increased from ~20% at 10 truncated points up to 70% at 30–40 points, while the Glu CoV rose from ~4% to 10%. A similar trend was observed for 10‐ and 11‐component models, where Glu bias increased from ~14% to 36% and CoV from ~6% to 10%.

tCho showed a similar but smaller effect: Bias increased from < 1% (10 points) to ~3% (30+ points), and CoV from 5% to 7%. In contrast, tCr and tNAA biases and CoVs remained relatively stable across truncation strategies (10‐ and 11‐component models: tCr bias ~1.6%–3%, tNAA bias ~1.7%–2%; CoV tCr ~4%–5%, CoV tNAA ~3%–4%).

Detailed results on truncation strategies using 15–40 points are provided in Figures S10–S13. Our findings highlight that excessive time‐point truncation can affect metabolite quantification and increase estimation variability and need to be chosen carefully.

**FIGURE 6:**
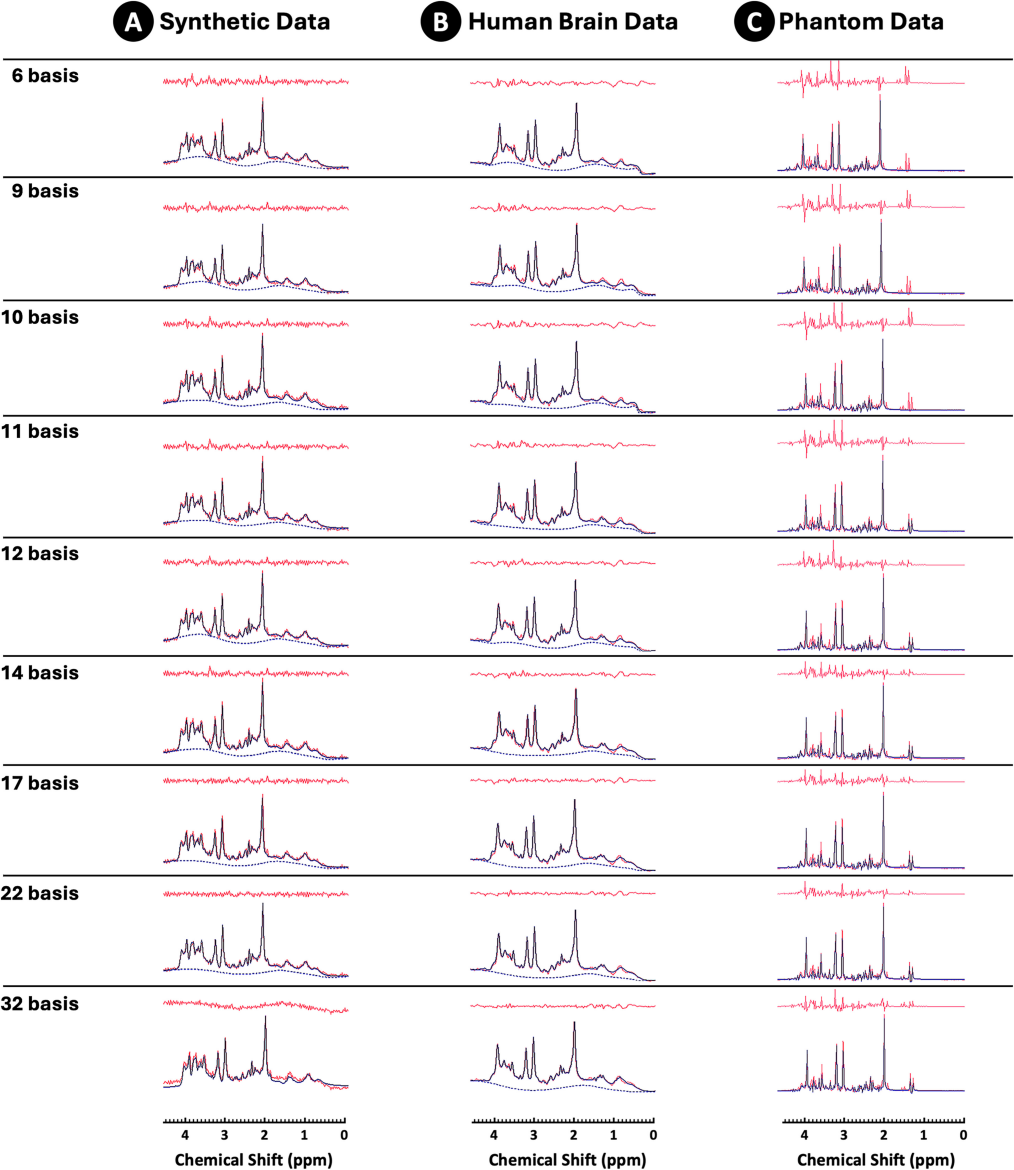
Synthetic, human brain and phantom spectra. Example of model fits (blue) shown together with data (red) for (A) synthetic human brain data (SNR 70), (B) in vivo and (C) phantom data (4‐min scanning duration) fitted with nine optimal basis set models. QUEST‐subtract background handling with 10 truncated points was used on synthetic and in vivo data. See Table 1 for the full breakdown of basis sets components.

Moreover, a visual inspection of the model fitting results and residuals (Figure 6A showing SNR 70 example using 10 truncated points) suggests that a better fit is observed with larger basis set models containing 17+ metabolite components. However, the comparison of nine optimal basis set models (Table 1) revealed that the 10‐ and 11‐component models (‘Base model + GABA, NAAG, GSH, Glc ± Lac’) performed better across all noise levels. Lower bias was observed for Glu (14%–17% vs. 20%–45%), tCho (0.47%–2.13% vs −3.38% to 9.83%) and tNAA (−1.68% to −1.98% vs. −1.82% to −4.49%) compared to the remaining models. For tCr, the results indicate a lower bias with a 6‐components base model compared to the 10‐ and 11‐component models (~0% vs. ~ −2%). Data variability remained relatively stable across all nine investigated models, staying within 10% of the mean. A negative relationship between data variability and SNR levels was observed, with variability decreasing as SNR increased (Figure 5).

Overall, these results indicate that basis models with 10 to 11 components (‘Base model + GABA, NAAG, GSH, Glc ± Lac’) can quantify the data as reliably as fully specified models. These findings align with the results from the previous model selection section, where we explored increasing noise levels without the added complexity of the BGMM handling, which is always present in in vivo data.

### 3.2 Human Brain Results

The model fitting results for a 4‐min scanning duration are shown in Figure 6B. Similar to the synthetic data fits, visual inspection of the measured human brain model fits and residuals suggests that larger basis set models (17+ components) provide better fits to the data. The background signal with a flatter baseline appears consistent across all nine basis set models, indicating that QUEST‐Subtract with 10 truncated points handled it effectively (Figure 6B). However, while adding more components to the model improves the fit and reduces residuals, it may also introduce potential overfitting and bias.

**FIGURE 7:**
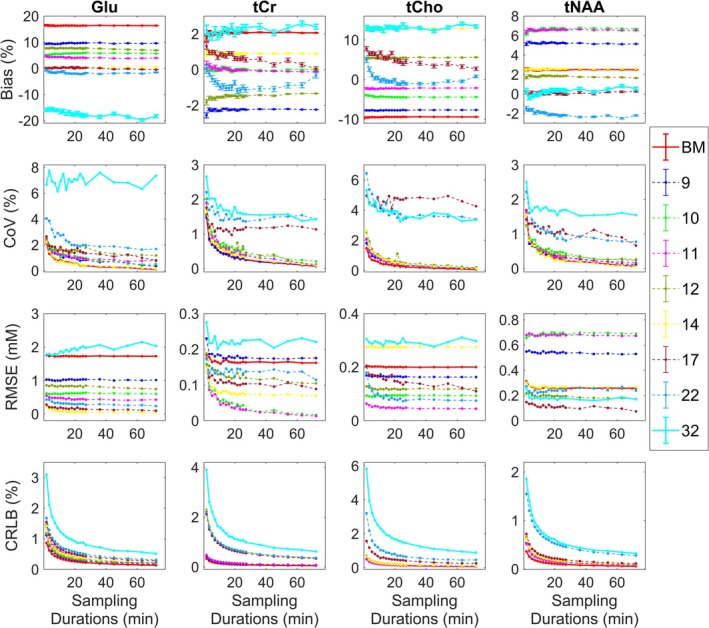
Bias, variance and fitting of human brain data. Results for the in vivo data analysis. Quantification of Glu, tCr, tCho and tNAA quantified with nine optimal basis sets over 20 durations (1.8–81 min). Top panel is showing bias from the ground truth (GT, %) with standard error bars, middle panel is showing variability in the data expressed as coefficient of variation (CoV, %), the third panel is showing root‐mean‐squared error (RMSE, mM) and the bottom panel is showing Cramér–Rao lower bound (CRLB, %) for metabolite estimates uncertainty. See Table 1 for the full breakdown of basis sets components. BM = base model.

Minimal basis set models with 10–11 components (‘Base model + GABA, NAAG, GSH, Glc ± Lac’) generally perform as well as fuller models across all sampled durations, as evident from Figure 7. The estimates for tCr are relatively stable, remaining within ±2% of the GT across all models. In contrast, tCho exhibits greater stability and precision with the 10–11 component models, particularly at lower SNR, as indicated by lower RMSE and CoV%. The basis model with 22 components performed best for longer durations, yielding the lowest bias but at a cost of higher CRLBs and variability compared to the 10–11 component models. tNAA quantification remained within ±6% of the GT, with fuller models yielding more accurate outcomes. Glu estimates, with the exception of the 6‐component base model and fully specified 32‐component model, stayed within 10% of the GT. Although models with 14+ components offer higher accuracy, the 10–11 component models achieved estimates within 4%–5% of the GT along with lower variability.

As seen in Figure 7, CRLBs of the estimates increase with a greater number of components in the basis sets, particularly when partner metabolites of tNAA, tCr and tCho are included. CRLBs are also higher for shorter acquisition durations with lower SNR (< 5 min) compared to longer durations (> 5 min) with higher SNR. As expected, the short acquisition durations (< 5 min) show larger variability and greater bias, whereas longer durations (> 5 min) show more stability in fitted parameters (Figure 7). Moreover, minimal basis sets (< 17 components) show lower variance at realistic scanning durations (< 5 min) compared to fully specified models, suggesting they provide more precise estimates. While a reduction in bias with minimal basis models with 10–11 components in some cases indicates improved accuracy over fuller models, the lack of a reliable GT in this analysis limits the generalizability of these results.

Concentrations of Glu, tCr, tCho and tNAA across all analyses were within the in vivo physiological range [41], showing reduction in values with fuller models for Glu (8.91–12.28 mM) and increase in values for tCho (1.92–2.43 mM). tCr (~7.6–8 mM) and tNAA (~10–11 mM) estimates remained reasonably stable across all fittings. Base model with six components showed higher Glu concentrations (12.28 mM), which were reasonably stable across all durations, whereas the full 32 signal model showed lower Glu concentration estimates (8.91 mM) compared to the rest of the models. This suggests an effect of basis set components on the quantification of Glu.

### 3.3 Phantom Results

**FIGURE 8:**
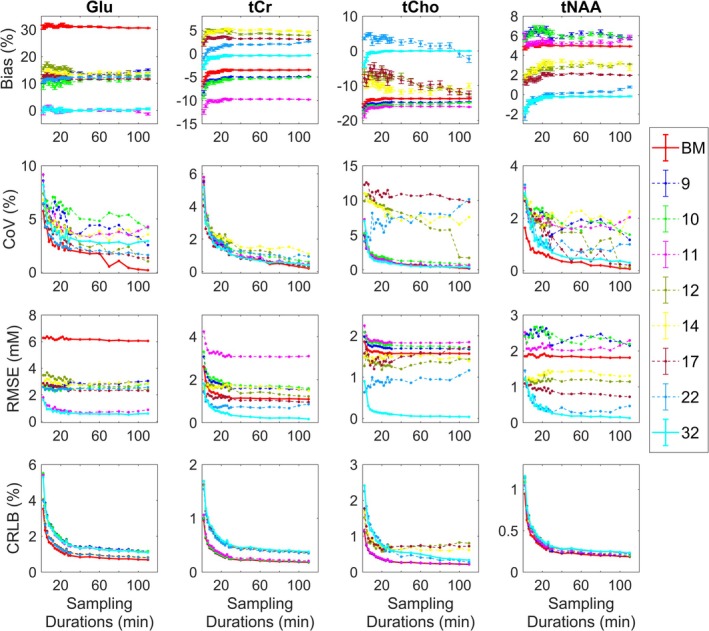
Bias, variance and fitting of phantom data. Results for the phantom data analysis. Quantification of Glu, tCr, tCho and tNAA quantified with nine optimal basis sets over 20 durations (2–120 min). Top panel is showing bias from the ground truth (GT, %) with standard error bars, middle panel is showing variability in the data expressed as coefficient of variation (CoV, %), the third panel is showing root‐mean‐squared error (RMSE, mM) and the bottom panel is showing Cramér–Rao lower bound (CRLB, %) for metabolite estimates uncertainty. See Table 1 for the full breakdown of basis sets components. BM = base model.

The model fitting results for a 4‐min scanning duration are shown in Figure 6C. As expected, visual inspection of the plots and residuals suggests that the better fit of the model is observed with the larger basis sets containing 22 and 32 metabolite components. Since the GT for this analysis is assumed to be the result of fitting the full acquisition duration (120 min) with a 32‐component basis model, corrected for metabolite and water T_2_ and T_1_ relaxation times, the fitting estimates for Glu, tNAA, tCr and tCho using the 32‐component model closely approximate the GT for durations longer than 5 min (high SNR). However, noticeable bias is present in estimates for durations shorter than 5 min (lower SNR) (Figure 8).

The results show that CRLBs are lower for analyses with minimal basis sets (6–11 components) across sampled durations. Basis sets with 10–11 components (‘Base model + GABA, NAAG, GSH, Glc ± Lac’) largely outperform basis sets with 12+ components for Glu quantification (Figure 8). tNAA and tCr estimates are within ±10% of the GT, with the variability ~0.5%–6%, apart from tCr estimated with an 11‐component model resulting in bias of ~ −13% in short scanning durations (< 5 min). More reliable and accurate estimates for tCho were produced with the models with 12+ components. CRLBs of the estimates largely increase with a greater number of components in the basis sets, particularly when partner metabolites of tCr and tCho are included. This is less evident for tNAA as the NAAG component was added to eight out of nine models. CRLBs are also higher in shorter durations and are reduced in durations > 5 min. The shorter durations (< 5 min) show larger variability and greater bias, whereas longer durations (> 5 min) show more stability in fitted parameters (Figure 8). Minimal basis models (< 12‐components) demonstrate mostly lower variability for all metabolites at realistic scan durations (< 5 min), indicating higher precision in estimates compared to fully specified models. However, for metabolites such as tCr, tCho and tNAA, minimal models exhibit greater bias, suggesting that fuller models may provide better accuracy for these metabolites. Notably, this increased bias was not observed for Glu.

Phantom T_1_ and T_2_ corrected concentrations of Glu, tCr, tCho and tNAA vary across all nine model fittings, exhibiting more stability in estimates with basis sets with lower number of components (< 14) (see Figure S2). Glu and tNAA show reduction in values with fuller models (20–26 and 36–39 mM), whereas there is an increase in values for tCho (9.5–12 mM) and tCr (28–33 mM) estimates. The 6‐component base model showed higher Glu concentrations (26 mM) and ~30% bias, which is consistent with the synthetic data analysis demonstrating similar results. Glu quantification with 11‐component model showed a bias of approximately 0.2%, matching our synthetic data estimates with the same basis model.

## 4 Discussion

This work examined the bias–variance trade‐off under different SNR conditions using synthetic data (with and without background and MM signals) as well as phantom and in vivo human brain data, both acquired at 3 T. Synthetic data with absolute GT enabled the identification of basis sets that minimised both bias and variance, which informed the subsequent analyses of the in vivo and phantom data. Phantom experiments validated these basis sets against a complex metabolite solution with known GT, while in vivo data provided a performance evaluation on biological spectrum.

Our findings indicate that models with fewer than 16 components are sufficient for accurate and precise quantification of tCr and tNAA across all three approaches. Optimal models for Glu include 10–11 components (base model + ‘GABA, NAAG, GSH, Glc, ± Lac’). However, tCho quantification remained inconsistent across three methods. Table 2 summarises the assessment metrics for the 10‐ and 11‐component models across four experimental approaches.

**TABLE 2:** Assessment metric results for metabolites of interest using 10‐ and 11‐component models.

Condition	Metric	Model with 10 components				Model with 11 components			
**Synthetic**		Glu_10_	tCr_10_	tCho_10_	tNAA_10_	Glu_11_	tCr_11_	tCho_11_	tNAA_11_
	Bias (%)	10.17	2.71	14.37	4.19	10.50	2.50	14.04	3.96
	CoV (%)	6.43	2.97	3.27	2.07	6.38	2.97	3.29	2.09
	RMSE (mM)	1.49	0.33	0.25	0.61	1.52	0.31	0.25	0.59
	CRLB (%)	6.69	2.63	3.55	4.13	6.66	2.63	3.56	4.10
**BGMM**	Bias (%)	14.83	−2.98	0.47	−1.98	14.83	−2.70	1.21	−1.88
	CoV (%)	6.45	3.89	5.14	2.45	6.55	4.00	5.36	2.42
	RMSE (mM)	2.00	0.38	0.09	0.40	2.00	0.38	0.09	0.39
	CRLB (%)	6.33	2.64	4.02	4.25	6.38	2.65	3.99	4.27
**In vivo**	Bias (%)	5.18	0.08	−3.91	6.17	4.60	0.28	−2.19	6.36
	CoV (%)	2.64	2.02	2.11	1.54	2.36	1.90	2.07	1.64
	RMSE (mM)	0.62	0.16	0.09	0.66	0.55	0.15	0.06	0.68
	CRLB (%)	1.40	0.48	0.51	0.52	1.46	0.48	0.50	0.52
**Phantom**	Bias (%)	8.72	−9.04	−17.74	5.78	−0.76	−12.45	−18.70	5.97
	CoV (%)	9.14	5.79	5.26	2.86	9.17	5.81	7.28	3.16
	RMSE (mM)	2.62	3.29	2.09	2.41	1.81	4.23	2.24	2.52
	CRLB (%)	5.52	1.01	1.18	1.08	5.40	1.06	1.17	1.05

*Note:* Results for synthetic and BGMM data are shown for SNR 35 condition. Results for In vivo and phantom data are shown for a 2‐min acquisition duration; see Table 1 for the full breakdown of components for each model.

Abbreviations: BGMM, background and macromolecules; CoV (%), coefficient of variation; CRLB (%), Cramér–Rao lower bound; Glu_10_ /Glu_11_, glutamate fitted with 10‐ and 11‐component model; RMSE (mM), root‐mean‐squared error; tCho_10_/tCho_11_, total choline fitted with 10‐ and 11‐component model; tCr_10_/tCr_11_, total creatine fitted with 10‐ and 11‐component model; tNAA_10_/tNAA_11_, total N‐acetylaspartate fitted with 10‐ and 11‐component model.

### 4.1 Metabolites of Interest

#### 4.1.1 Closely Related Metabolites of tNAA, tCr and tCho

Metabolites such as Cr and PCr as well as NAA and its derivative NAAG exhibit highly similar spectra, with negligible chemical shift differences (< 0.015 ppm), making it impossible to resolve their respective resonances at field strengths ≤ 3 T [2, 41]. As a result, their combined signals (Cr + PCr, NAA + NAAG) are often used as representative markers of Cr‐ and NAA‐containing compounds. The tCho signal appears as a prominent singlet at 3.2 ppm, originating from multiple choline‐containing compounds: GPC, PCh and free Cho. Due to the small chemical shift difference, the methyl protons of PCh and GPC are difficult to separate, and free Cho is undetectable with standard MRS [41, 42] The combined signals of tCr, tNAA and tCho contribute significantly to the overall spectrum.

A priori, it might appear that incorporating as much prior information in a model as is available should lead to better outcomes. However, this assumption only holds true in the absence of noise. This is clearly illustrated in the synthetic spectra, where, for example, inclusion of both NAAG and PCr does not significantly reduce the bias or variance of fitting tNAA and tCr. While tCho quantification benefited from PCh/PCh + Cho inclusion in larger models, reduced models (10–11 components: the six base metabolites (Cr, Glu, Gln, GPC, m‐Ins, NAA) + ‘GABA, NAAG, GSH, Glc, ± Lac’) yielded results as reliable as fully specified models for this metabolite (Figure 4).

Similarly, in our in vivo and phantom studies, the inclusion of PCr and NAAG components did not contribute to reduction in bias and variance in fitting tNAA and tCr. On the contrary, adding extra components to the basis model led to a noticeable increase in variance, particularly evident for short scanning durations (< 5 min) (Figures 7 and 8). As with the synthetic data, tCho quantification in both human and phantom studies appeared to benefit from including PCh or PCh + Cho in the model. However, for human brain data, reduced models (10–11 components: base model + ‘GABA, NAAG, GSH, Glc, ± Lac’) produced results as reliable as fully specified models, with a lower variance and bias below 5% of the GT (Figure 7).

We note that metabolite concentration estimates varied across the nine basis set fittings in both the human brain and phantom studies. The in vivo concentrations for tCr and tNAA were within physiological range for a healthy brain (8–10 and 8–11 mM, respectively) [42, 61] and reasonably stable across all nine basis sets and all durations. The concentrations for tCho noticeably differed across all model fittings, depending on the metabolite components included in the basis sets. Basis models with only one metabolite component for tCho (e.g., GPC) yielded more plausible concentration estimates (1.93–2.08 mM) in line with physiological range for a healthy brain (1–2 mM) [42, 61], whereas basis models with more than one component representing tCho signal yielded much higher values (2.23–2.43 mM). Consistent with the in vivo analysis, tCho estimates in the phantom also varied depending on the components used, with fuller models producing higher estimates (~12 mM).

Our findings are somewhat consistent with Hofmann and colleagues [27] who also investigated the inclusion of closely related metabolites in basis sets. Similar to our results, they reported that including partner metabolites for tCr, tNAA and tCho changes both the estimated content of these metabolites and the quantification of others in the spectrum [27]. Particularly compelling evidence was presented for choline‐containing compounds, where modelling Cho as a single component (e.g., Cho) instead of Cho ⫹ PCh ⫹ GPC caused significant differences in quantification outcomes as in the present study. For tNAA and tCr, our findings suggest that including NAAG and PCr in the model does not significantly improve their estimates. However, Hofmann et al. [27] argued that if the standard deviations of metabolites change only slightly with additional components and their estimated concentrations exceed the standard error of the mean, it is acceptable to include more metabolites in the basis model. Furthermore, a novel data‐driven basis set optimisation approach demonstrated by Davies‐Jenkins et al. [40] provides additional support for our findings. Authors illustrated that all basis models (variations of 1–32 metabolite components) derived from the simulated data correctly identified high‐concentration metabolites (e.g., tNAA, tCr, tCho and Glx) with bias within 10% of the GT and provided reasonable fits of the spectra [40].

#### 4.1.2 Glutamate Quantification

Glutamate is the most abundant amino acid in the brain, reaching concentrations up to 12 mM [2]. As the principal excitatory neurotransmitter [2], it is widely studied in MRS research. While including metabolites like NAAG and PCr showed little benefit for quantifying their respective partner metabolites (NAA and Cr), our analysis of synthetic spectra (Figure 4) revealed that including GABA, NAAG and GSH in the basis set significantly reduces bias in Glu estimation.

Unlike the spectral profiles of partner metabolites, Glu is located in a crowded spectroscopic region, sharing multiple overlapping resonances with NAAG, GSH, Gln and GABA. These overlaps can result in systematic bias if not accounted for in the model. NAAG, for example, has a very similar spectrum with the N‐methyl group resonating at 2.05 ppm in the same region as the Glu C3 protons and the Glu moiety of NAAG shifted by only 0.05–0.1 ppm relative to Glu itself. This can potentially lead to misattribution of signals if NAAG is not included in the model. Similarly, GABA and GSH overlap with Glu around 2.1–2.3 ppm [41, 42]. Our data show that omitting any of these metabolites results in increased bias and variance in Glu quantification, highlighting the necessity of including all three to obtain reliable Glu estimates. However, we note that expanding the basis set beyond 12+ metabolic components did not yield further improvements in Glu quantification. In fact, it often increased variance, especially under low‐SNR conditions (synthetic spectra) or with shorter scanning durations (phantom and in vivo studies).

Our human brain and phantom studies further support these findings. As with tNAA, tCr and tCho quantification, Glu concentrations also varied across the nine evaluated basis sets. Using a 6‐metabolite base model that excluded GABA, NAAG and GSH led to overestimated Glu levels (~26 mM in phantom data/GT ~20 mM; ~12.28 mM in vivo data/GT ~10.55 mM) and greater bias (~30% in phantom; ~16% in vivo). These results align with our synthetic brain spectra, which also showed elevated Glu bias when using the base model. Moreover, the 6‐metabolite base model produced concentrations near the upper limit of the typical physiological range for a healthy brain (6–12 mM) [2], whereas the remaining eight models produced concentration estimates within the expected range (~8.91–10 mM).

Our findings are also in line with a recent work by Demler et al., who observed variations in Glu concentrations across five basis sets (15–19 components) commonly used in psychosis research [30]. They reported more consistent results with basis sets that included the PCr component. While we also observed differences in Glu quantification across the nine basis models evaluated in our study, our results suggest that models both with and without the PCr component produced reasonable quantification outcomes across scanning durations. The discrepancies between our findings and those of Demler et al. [30] may be explained by several methodological differences: variations in number and composition of basis set components explored (15–19 components vs. 6–32 components in our study), use of different packages for basis set simulations (Magnetic Resonance Spectrum Simulator [MARSS] [19] vs. NMRScopeB [43] in our study) and lastly, use of different fitting and quantification toolboxes (Spectroscopy Analysis Tools [spant] [62] and LCModel implemented in Osprey [11] vs. jMRUI QUEST [13] in our study). All these differences highlight the impact of methodological choices on Glu quantification outcomes and emphasise the need for standardised approaches in future MRS studies.

### 4.2 Basis Set Selection

There is no consensus in the MR community on which metabolites should be included in the linear combination of basis spectra when fitting ^1^H‐MR spectra. Often, over 16+ metabolite signals are included in the basis models [10, 11, 26, 30, 31, 33]. However, it is not clear if fuller models are beneficial at 3 T.

Existing literature demonstrates a strong dependence of metabolite detectability and quantification accuracy on magnetic field strength. At clinical field strengths (1.5–3 T), the total signals of tNAA, tCr and tCho can be reliably detected [2, 41], whereas their partner metabolites NAAG, PCr, PCh/Cho, respectively, cannot be reliably resolved at ≤ 3 T due to very small chemical shift differences between them [41, 42]. *Myo*‐Ins, the most abundant inositol isomer, is detectable at ≥ 1.5 T with improved separation from Glc and Gly at 3 T [61, 63]. *Scyllo*‐Ins, the second most abundant isomer, has been observed at ≥ 2 T [64, 65]. At intermediate to high fields (3–7 T), greater spectral resolution improves the separation of J‐coupled metabolites. Glu and Gln become partially resolved at 3 T and clearly distinct at ≥ 7 T, substantially improving quantification [32, 66]. GABA multiplets in the 1.9–3.0 ppm range overlap with Glu, tCr, tNAA, GSH and MM resonances, requiring spectral‐editing methods at 3 T (e.g., MEGA‐PRESS) for accurate quantification and allowing direct fitting only at ≥ 7 T [35, 67, 68, 69]. GSH detection similarly relies on editing at 3 T [24, 70, 71] but can be quantified directly at ≥ 4 T [34, 72].

Given these magnetic field–dependent detectability constraints, we set out to examine how basis set composition influences the quantification of four key metabolites: tNAA, tCr, tCho and Glu at 3 T. As evident from Figures 4, 7 and 8, all three of our methodological approaches consistently showed that reduced basis sets containing between 10 and 11 components (base model + ‘GABA, NAAG, GSH, Glc, ± Lac’) performed better at detecting changes in these metabolites than fully specified models with 17+ components (Table 1). Clearly, our data allow us to interrogate bias/variance over a range of SNR for other important metabolites, such as GSH, GABA and m‐Ins, but in the interests of brevity, we confined our analysis to the above four. However, generalised conclusions can be drawn for the other metabolites based on SNR and signal complexity—lower SNR and increased J‐coupling will tend to increase bias and variance, which, for variance, will be more pronounced for larger basis sets.

Using synthetic, human brain and phantom spectra, we demonstrate that adding metabolites with physiological concentrations above 0.5 mM (NAAG, PCr, PCh, GABA, Lac, GSH, Asp, Glc, s‐Ins and Gly) or below 0.5 mM (for basis models > 16 components, Table 1) to the base model (‘Cr, Glu, NAA, GPC, Gln, m‐Ins’) had minimal impact on tNAA and tCr quantification. Only a small benefit was observed for tCho estimates. These results are consistent with findings by Hofmann et al. [27], who showed that low‐concentration, non‐overlapping metabolites (e.g., Thr) do not significantly influence the estimation of well‐represented metabolites such as tNAA, tCr and tCho. For Glu, however, including low‐concentration metabolites with minimal spectral overlap (e.g., Thr and s‐Ins) led to an increase in variance and less accurate estimates, particularly under low‐SNR conditions. In contrast, including metabolites with strong spectral overlap in the crowded 2–3‐ppm region (e.g., GABA, NAAG and GSH) had a more pronounced effect on Glu quantification.

Furthermore, from our full analysis, it is evident that the precise choice of metabolites to be included in basis sets with more than nine components has relatively little effect on the performance of the algorithms for measuring the four target metabolites. For example, adding either Tau or s‐Ins to an 11‐signal basis set model has virtually no effect on the bias/variance of Glu, tCr, tCho and tNAA.

As expected, we show that fitted parameters in the low‐SNR conditions and short scanning durations (< 5 min) have larger variability and less accuracy in estimates. The CRLBs of the estimates for all four metabolites of interest largely increased with a greater number of components in the basis sets and for durations < 5 min, particularly when partner metabolites of tNAA, tCr and tCho were included in the models.

Our findings highlight the inherent compromise between accuracy (bias) and precision (variability) in the presence of noise when selecting an appropriate basis model. Increasing model complexity by including low‐concentration metabolites introduces additional parameters, while excluding such metabolites (e.g., PCh, Cho in 10–11 component models) results in negligible bias and reduced variance for well‐represented metabolites such as Glu, tNAA, tCr and tCho.

Finally, in this work, all analyses were conducted in the time domain using the QUEST algorithm implemented in jMRUI. Our approach, based on bias–variance trade‐off optimisation, is largely model‐independent with respect to the fitting domain. According to the recent ISMRM'18 MRS Fitting Challenge [17], both LCModel (frequency domain) and QUEST (time domain) ranked among the top‐performing algorithms, producing comparable quantification results. In addition, a recent data‐driven basis set optimisation study conducted in the frequency domain [40] reported outcomes consistent with our work, where tNAA, tCr, tCho and Glx were all quantified with bias within 10% of the GT. Consequently, we would expect our findings to be applicable across both fitting domains.

### 4.3 Inclusion of Baseline and Macromolecules

The background signal in ^1^H‐MRS spectra is usually considered a ‘nuisance’ parameter that must be accounted for to obtain reliable estimates for the metabolites of interest. A large part of the background signal consists of macromolecules with smaller contributions from the fast‐decaying baseline [25]. The ‘nuisance’ signal can be modelled with a finite number of parameters, using wavelets [73], splines (LCModel [10], AQSES [14]), a sum of exponentially damped sinusoids (QUEST [13, 52]) or by including a measured in vivo MM signal in the basis sets [7].

In this work, we used QUEST‐Subtract [52] to model baseline components, and nine optimal basis sets containing a measured in vivo MM spectrum to account for all MM resonances present at 3 T. It is clear from our BGMM results that truncating ≥ 20 ms of the FID leads to increased variability and overestimations in concentrations for all metabolites of interest (see Figures S10–S13). These findings are consistent with Starčuková et al.'s work [25] suggesting that in QUEST, the ‘subtract’ option should only be applied within 10 ms when using basis sets without measured MM signals. Since our basis sets already incorporate MM components through a composite MM spectrum, we found that truncating more than 5 ms of the FID increased variability and fitting uncertainty. This suggests that more conservative truncation is necessary when MM signals are explicitly modelled.

Moreover, visual inspection of the model fits and residuals (Figure 6A,B) indicates that the background signal with a flatter baseline appears consistent across all nine basis set models in both BGMM and in vivo data analyses, indicating that QUEST‐Subtract with 10 truncated points handled it effectively. This suggests that baseline modelling is not the primary source of residuals. Moving from 6 to 10 and up to 12 component models reduces residuals, but the baseline shape itself remains largely unchanged. With models larger than 12 components, residual noise decreases slightly and then plateaus, again without altering the baseline appearance.

While the 22‐ and 32‐component models maintain low residuals, the larger number of components increases the risk of overfitting and uncertainty (Figures 5 and 7). Notably, these models include threonine (22‐component model) and tyrosine + valine (32‐component model), which are recognised as MM peaks [7], and the question arises as to whether there could be cross‐talk between these amino acid residues and the underlying MM signal. However, linewidths were constrained to be Lorentzian and equal for all metabolite components, whereas a much broader Gaussian lineshape was assumed for the MM baseline, which was treated as a single component. Thus, the inclusion of these additional amino acid resonances in the basis sets will have no influence on fitting the MM component.

Furthermore, as with our other approaches, we found that basis models with 10 to 11 components (base model + ‘GABA, NAAG, GSH, Glc, ± Lac’) quantified the BGMM synthetic data as reliably as fully specified models across all noise levels. Both variability and CRLBs showed a clear relationship with noise levels and the number of components in the basis set (Figure 5). Notably, the inclusion of BGMM patterns significantly improved tCr and tNAA quantification, with an even more pronounced effect on tCho, reducing bias to below 10% of the GT. In contrast, there was no clear benefit for Glu quantification, highlighting the inherent sensitivity of J‐coupled metabolites to background signal contributions.

Lastly, in this study, we modelled the measured, parameterised noise‐free MM spectrum as a single component, fitted simultaneously with the metabolite basis set in the QUEST nonlinear least‐squares routine. Alternative MM modelling approaches, for example, representing MMs as multiple parameterised peaks, should be explored to test the robustness of our findings. Such models could change the relative MM contribution to the fit, increase the number of free parameters and consequently raise fitting uncertainty. Nonetheless, because our basis set optimisation is model‐independent and framed in terms of minimising bias–variance trade‐offs under realistic SNR conditions, we expect the overall trends and conclusions to remain valid.

### 4.4 Limitations and Future Directions

Several limitations of our three approaches should be acknowledged. For in silico simulations, we focused exclusively on synthetic data representing a healthy brain under ideal conditions. Future work could expand this approach to include metabolite components relevant to specific clinical conditions, which are not present in the healthy brain (e.g., brain tumour‐related metabolites such as 2‐hydroxyglutarate [74]). In addition, we will address potential acquisition artefacts commonly seen in MRS spectra, such as spurious echoes, residual water signals and correlated noise [75]. Moreover, metabolite LW significantly influences quantification accuracy, especially for complex spin systems such as Glu. As our simulations used 5 Hz line broadening, the results may not fully reflect in vivo LW variations (often > 7–10 Hz). However, following some simulations (not reported here), it is apparent that bias is relatively unaffected by an increase in metabolite LW to 10 or 15 Hz, whereas variance increases significantly for both simple and complex spin systems. The consequence of this is that our simulation results will underestimate variance expected for LW of 10 Hz or greater, but this does not affect the conclusion that reduced basis sets will perform better. Perhaps the main consequence of broader lines is reduced SNR, and we have shown that at low SNR the case for reduced basis sets is strongest.

For in vivo data, the main limitation is the absence of a true GT. While fitting the full acquisition duration (81 min) with the 17‐component basis set model serves as a reasonable alternative, it remains unclear whether this approach represents the most suitable GT for all fittings. At short TEs, a background signal, even if it is considered as a single MM composite component, increases the metabolite fitting uncertainties (CRLBs) and hinders the quantification. This is particularly evident for low‐concentration metabolites [25]. There is a trade‐off between a good fit of the background signal and a good fit of the metabolite signal due to the strongly overlapping metabolite patterns and MM background signal. In the presence of noise, strongly correlated parameters cannot be reliably retrieved and decorrelated by any quantification algorithm [25]. Employing various quantification toolboxes and background handling methods could provide additional insights into how different fitting methods influence results.

To mitigate the lack of GT in in vivo acquisitions, we designed the in vitro phantom experiment, where the exact quantities of each metabolite are known. While these concentrations at the point of phantom creation (Table S1) could, in principle, be used as the GT, they would need to be converted to signal amplitudes by correcting for metabolite T_1_ and T_2_ relaxation. We did not calculate relaxation times for the low‐concentration metabolites because the SNR was too low and therefore had no reliable way to apply these corrections. Using an 800 MHz spectrometer enabled us to establish if any degradation occurred over a 5‐h acquisition time, indicating negligible concentration changes due to small frequency drifts. However, the 800 MHz data could not be used as a quantitative reference either, as the ultra‐high‐field measurements suffered from alignment and quantification limitations. Furthermore, the MRS signal amplitude depends on T_2_, T_1_ and the acquisition parameters. Measurement of relaxation times will introduce errors that feed into signal amplitude estimates, so we considered the signal amplitudes averaged over the full 120‐min acquisition as the best approximation to GT and then converted the results into mM units using the water signal and the measured relaxation times of water and metabolites. Notably, while a phantom solution does not have the complexities of the background signal, a baseline noise level is still present in the measurements and contributes to variability in the data.

Finally, we restricted our analyses to Glu, tCr, tCho and tNAA to emphasise metabolites that are robustly quantified in unedited short‐TE ^1^H‐MRS. While other metabolites such as GABA, m‐Ins, GSH and Gln are of great interest to the MRS community, the four we have chosen act as exemplars of both simple and complex spin systems, allowing generalisation of our conclusions to other metabolites. We demonstrate that reduced basis sets give optimal bias/variance performance and that this is more pronounced at low SNR and for more complex spin systems such as Glu, compared to tNAA or tCr. Therefore, quantification of molecules present at lower concentrations, such as GABA and GSH, will benefit more from reduced basis sets. However, due to concerns of brevity, we have not expanded the analyses to include other metabolites.

## 5 Conclusions

We show that tCr, tNAA, tCho and Glu can be reliably quantified across all three methods using minimal basis models with 10–11 components, consisting of six base metabolites (‘Cr, Glu, Gln, GPC, m‐Ins, NAA’) plus ‘GABA, NAAG, GSH, Glc, ± Lac’. Including partner metabolites for tCr and tNAA (e.g., PCr and NAAG) did not improve outcomes for these metabolites. While tCho quantification benefited from PCh inclusion in larger models, minimal models (10–11 components) yielded results as reliable as fully specified models. Glu quantification significantly improved when ‘GABA, NAAG, GSH ± Lac/Glc’ were added to the model. Therefore, we recommend that at the least ‘GABA, NAAG and GSH’ should be added to the minimal basis set models for more reliable Glu estimates.

Metabolite quantification balances bias and variance. Including more metabolites than necessary, particularly low concentration or weakly represented ones, increases model complexity, leading to higher variance, greater fitting uncertainty and an increased risk of overfitting (particularly at low SNR). By contrast, omitting these metabolites introduces only minimal bias in the estimation of well‐represented signals. Consequently, the number of metabolite components that can be modelled reliably is SNR‐dependent: High‐SNR spectra can accommodate more components without overfitting, which is a risk in low‐SNR data. The practical consequence of including more metabolites than supported by the data is that statistically significant metabolite difference/change may be missed because of the increased variance in the estimates. This study demonstrates that variations in reported metabolite concentrations across literature are partly due to differences in the prior knowledge models used, even when the same fitting algorithm is applied. These findings highlight the importance of developing standardised analysis approaches to improve consistency and reproducibility in MRS research.

## Author Contributions

P.E. contributed to conceptualization, data acquisition, methodology, software, validation, formal analysis, investigation, visualisation, writing, review and editing, project administration. L.M.P. contributed to conceptualization, data acquisition, investigation, review and editing, supervision. C.L.‐C. contributed to conceptualization, data acquisition, software, investigation, review and editing, supervision. S.R.W. contributed to conceptualization, data acquisition, methodology, software, validation, formal analysis, investigation, visualisation, writing, review and editing, supervision.

## Conflicts of Interest

The authors declare no conflicts of interest.

## Supporting information


**Appendix S1:** Supporting information.
**Figure S1:** Model selection for synthetic data analysis using six prominent metabolites (Glu, Gln, NAA, Cr, m‐Ins and GPC) as a base model. Components between 1 and 10 (GABA, Lac, PCr, PCh, NAAG, GSH, Asp, Glc, s‐Ins and Gly) were sequentially added to the base model, creating models with up to 16 components. Models > 16 components were sources from the literature.
**Table S1:** Metabolite concentrations in the physiological range used in the brain‐mimicking phantom.
**Figure S2:** Phantom T1 and T2 corrected concentrations with standard error bars for Glu, tCr, tCho and tNAA quantified with nine optimal basis sets over 20 durations (see **Table 1** for metabolite components in each model). All metabolite concentration estimates show variability between nine fitted models. BM = base model.
**Figure S3:** Signal vs. TE data and fitted curves from which T2 estimates were extracted for Cr, PCr, NAA, NAAG, Glu and GPC in brain‐mimicking phantom acquired at 3 T.
**Figure S4:** Signal vs. TI data and fitted curves from which T1 measurements were extracted for Cr, NAA, NAAG, Glu and GPC in brain‐mimicking phantom acquired at 3 T.
**Figure S5:** Glu, tCr, tCho and tNAA quantification with ten 7‐component model variations. Top panel is showing bias from the ground truth (GT, %) with standard error bars, middle panel is showing variability in the data expressed as coefficient of variation (CoV, %), the third panel is showing root‐mean‐squared error (RMSE, mM) and the bottom panel is showing Cramér–Rao lower bound (CRLB, %) for metabolite estimates uncertainty. BM = base model with six components is shown in solid red line. Glu = glutamate, tCr = total creatine, tCho = total choline, tNAA = total N‐asetylasparate. BM = base model, Asp = aspartate, Gaba = γ‐aminobutyric acid, Glc = glucose, Gly = glycine, GSH = glutathione, Lac = lactate, NAAG = N‐asetylaspartylglutamate, PCh = phosphorylcholine, PCr = phosphocreatine, s‐Ins = scyllo‐inositol. See **Table 1** for the full breakdown of basis sets components.
**Figure S6:** Glu, tCr, tCho and tNAA quantification with nine 8‐component model variations. GABA component is present in all fittings. BM = base model with six components is shown in solid red line.
**Figure S7:** Glu, tCr, tCho and tNAA quantification with four 9‐component model variations. NAAG and GSH components are present in all fittings with GABA, Lac, PCh and PCr, respectively. BM = base model with six components is shown in solid red line.
**Figure S8:** Glu, tCr, tCho and tNAA quantification with ten 10‐component model variations. NAAG and GSH components are present in all fittings. BM = base model with six components is shown in solid red line. ‘G’ = GABA, NAAG and GSH were included in all models. ‘L’ = lactate, NAAG and GSH were included in all models. ‘PCrPCh’ = NAAG, GSH, PCr and PCh were included in all models.
**Figure S9:** Glu, tCr, tCho and tNAA quantification with nine 11‐component model variations. BM = base model with six components is shown in solid red line. G = ‘GABA NAAG GSH’ present in all models; GG = ‘GABA GSH LAC’ present in the model. GN = ‘GABA NAAG LAC’ present in the model. NG = ‘NAAG GSH LAC’ were included in the model.
**Figure S10:** Glu, tCr, tCho and tNAA quantification with nine optimal basis sets across seven SNR levels, using QUEST‐subtract background handling with 15 truncated points. Top panel is showing bias from the ground truth (GT, %) with standard error bars, middle panel is showing variability in the data expressed as coefficient of variation (CoV, %), the third panel is showing root‐mean‐squared error (RMSE, mM) and the bottom panel is showing Cramér–Rao lower bound (CRLB, %) for metabolite estimates uncertainty. BM = base model with six components is shown in solid red line. See **Table 1** for the full breakdown of basis sets components.
**Figure S11:** Glu, tCr, tCho and tNAA quantification with nine optimal basis sets across seven SNR levels, using QUEST‐subtract background handling with 20 truncated points. BM = base model with six components is shown in solid red line. See **Table 1** for the full breakdown of basis sets components.
**Figure S12:** Glu, tCr, tCho and tNAA quantification with nine optimal basis sets across seven SNR levels, using QUEST‐subtract background handling with 30 truncated points. BM = base model with six components is shown in solid red line. See **Table 1** for the full breakdown of basis sets components.
**Figure S13:** Glu, tCr, tCho and tNAA quantification with nine optimal basis sets across seven SNR levels, using QUEST‐subtract background handling with 40 truncated points. BM = base model with six components is shown in solid red line. See **Table 1** for the full breakdown of basis sets components.
**Table S2:** AMARES analysis results of first and last spectrometer acquisition.
**Figure S14:** Difference spectrum between first and last high‐resolution phantom acquisition on Bruker spectrometer at 800 MHz.

**Appendix S2:** MRSinMRS checklist.

## Data Availability

The data supporting findings of this study are available from the corresponding author upon reasonable request.
