# Supplementary material for: Influence of Basis Set Composition on Metabolite Quantification of 1H‐MRS at 3 T: Combining In Silico, In Vivo and In Vitro Evidence
Source: NMR Biomed. 2026 Feb 11;39(3):e70230. doi: 10.1002/nbm.70230 (PMC12894809; doi:10.1002/nbm.70230)
Supplement: Supplementary file 1 — Appendix S1: Supporting information. Figure S1: Model selection for synthetic data analysis using six prominent metabolites (Glu, Gln, NAA, Cr, m‐Ins and GPC) as a base model. Components between 1 and 10 (GABA, Lac, PCr, PCh, NAAG, GSH, Asp, Glc, s‐Ins and Gly) were sequentially added to the base model, creating models with up to 16 components. Models > 16 components were sources from the literature. Table S1: Metabolite concentrations in the physiological range used in the brain‐mimicking phantom. Figure S2: Phantom T1 and T2 corrected concentrations with standard error bars for Glu, tCr, tCho and tNAA quantified with nine optimal basis sets over 20 durations (see Table 1 for metabolite components in each model). All metabolite concentration estimates show variability between nine fitted models. BM = base model. Figure S3: Signal vs. TE data and fitted curves from which T2 estimates were extracted for Cr, PCr, NAA, NAAG, Glu and GPC in brain‐mimicking phantom acquired at 3 T. Figure S4: Signal vs. TI data and fitted curves from which T1 measurements were extracted for Cr, NAA, NAAG, Glu and GPC in brain‐mimicking phantom acquired at 3 T. Figure S5: Glu, tCr, tCho and tNAA quantification with ten 7‐component model variations. Top panel is showing bias from the ground truth (GT, %) with standard error bars, middle panel is showing variability in the data expressed as coefficient of variation (CoV, %), the third panel is showing root‐mean‐squared error (RMSE, mM) and the bottom panel is showing Cramér–Rao lower bound (CRLB, %) for metabolite estimates uncertainty. BM = base model with six components is shown in solid red line. Glu = glutamate, tCr = total creatine, tCho = total choline, tNAA = total N‐asetylasparate. BM = base model, Asp = aspartate, Gaba = γ‐aminobutyric acid, Glc = glucose, Gly = glycine, GSH = glutathione, Lac = lactate, NAAG = N‐asetylaspartylglutamate, PCh = phosphorylcholine, PCr = phosphocreatine, s‐Ins = scyllo‐inositol. See Table 1 for the fu [file NBM-39-e70230-s001.pdf]

## Appendix A. Supplementary Tables and Figures.

## Appendix A1. Model selection

Examples of basis set model compositions used in synthetic data analysis are presented in **Figure S1** below.

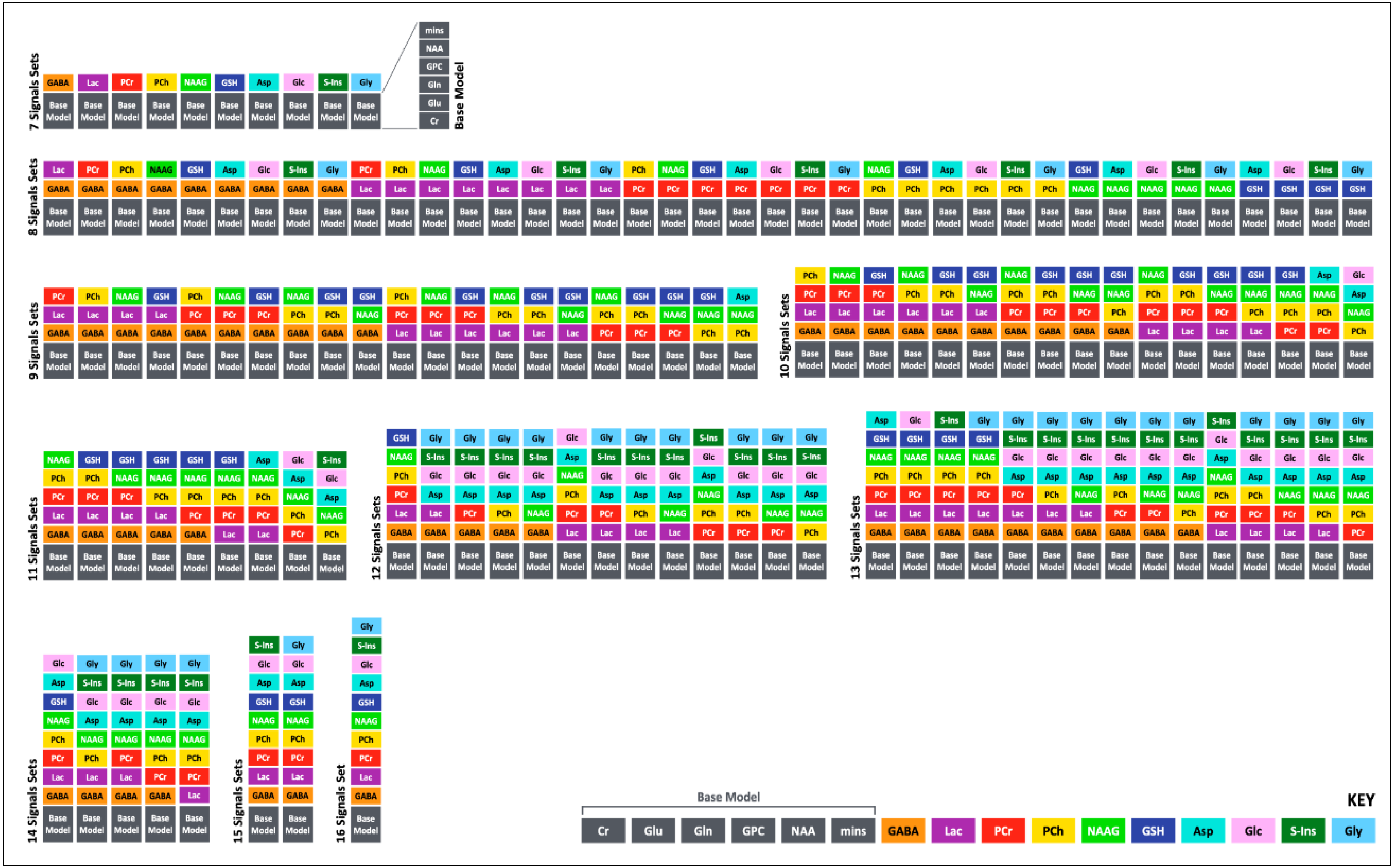


**Figure S1.** Model selection for synthetic data analysis using 6 prominent metabolites (Glu, Gln, NAA, Cr, m-Ins, GPC) as a base model. Components between 1 and 10 (GABA, Lac, PCr, PCh, NAAG, GSH, Asp, Glc, s-Ins, Gly) were sequentially added to the base model, creating models with up to 16 components. Models > 16 components were sources from the literature.

## Appendix A2. Phantom composition and quantification.

The brain-mimicking phantom, containing the brain's major metabolites is shown in the **Table S1** below. The concentrations calculated from the fitting with the 9 optimal basis set models over a range of durations (2 - 120 minutes), corrected for water and metabolite $T_1$ and $T_2$ relaxation times and converted into mM units, are shown in **Figure S2**. The $T_2$ and $T_1$ measurements for the studied metabolites are presented in **Figures S3** and **Figure S4** below. The metabolite data were fitted with nonlinear least-square algorithm ('lscurvefit') in Matlab.

**Table S1.** Metabolite concentrations in the physiological range used in the brain mimicking phantom.

| Chemical | Name | MW | Final Con (mM) |
|---|---|---|---|
| **Gold Standard Phantom** | | | |
| 1. NAA | N-Acetylaspartate | GSP | 31.25 |
| 2. GABA | γ-Aminobutyric Acid | GSP | 5.0 |
| 3. CHO | Choline chloride | GSP | 7.5 |
| 4. CR | Creatine | GSP | 25.0 |
| 5. GLU | Glutamate | GSP | 31.25 |
| 6. M-INS | *Myo*-inositol | GSP | 18.75 |
| 7. LAC | Lactate | GSP | 12.5 |
| **Additional metabolites** | | | |
| 8. ALA | L-Alanine | 139.58 | 2.39 |
| 9. ACE | Sodium acetate | 136.08 | 1.42 |
| 10. ASP | L-Aspartic acid sodium salt monohydrate | 173.11 | 2.42 |
| 11. CAR | L-Carnosine | 226.23 | 0.40 |
| 12. ETA | Ethanolamine hydrochloride | 97.55 | 5.97 |
| 13. GLC | D-(+)-Glucose, monohydrate | 198.18 | 1.80 |
| 14. GLN | L-Glutamine | 146.15 | 10.0 |
| 15. GLY | Glycine | 97.05 | 1.75 |
| 16. GPC* | sn-Glycero-3-phosphocholine | 257.22 | 1.74 |
| 17. GSH | L-Glutathione reduced | 307.33 | 3.58 |
| 18. HIS | L-Histidine monohydrochloride monohydrate | 209.63 | 0.16 |
| 19. NAAG | N-acetyl Asp Glu | 304.25 | 4.07 |
| 20. PCH* | Phosphocholine chloride Sodium salt | 263.57 | 1.01 |
| 21. PCR | Creatine phosphate disodium salt | 255.08 | 9.57 |
| 22. PE | O-Phosphorylethanolamine | 141.06 | 2.64 |
| 23. PHE | L-Phenylalanine methyl ester hydrochloride | 215.68 | 0.19 |
| 24. PYR | Sodium pyruvate | 110.04 | 0.40 |
| 25. SER | L-Serine methyl ester hydrochloride | 155.58 | 0.73 |
| 26. SUC | Sodium succinate dibasic hexahydrate | 270.14 | 0.69 |
| 27. S-INS* | Scyllo-inositol | 180.16 | 1.06 |
| 28. TAU | Taurine | 125.15 | 2.61 |
| 29. THR | L-Threonine methyl ester hydrochloride | 169.61 | 0.51 |
| 30. TRY | L-Tryptophan ethyl ester hydrochloride | 268.74 | 0.19 |
| 31. TYR | L-Tyrosine disodium salt hydrate | 225.15 | 0.19 |
| 32. VAL | L-Valine methyl ester hydrochloride | 167.63 | 0.17 |

**Note.** Values shown are 5 x the physiological concentrations for a higher signal to noise (SNR) ratio. Final concentration values indicate the combined 400 ml GSP solution with 400 ml of additional metabolites solution (pH = 7.3). MW = molecular weight. GSP = Gold Standard Phantom with added Gd-DTPA. *Chemicals acquired from BOC Sciences, USA, the rest are from the Glentham Life Sciences (GLS), UK.

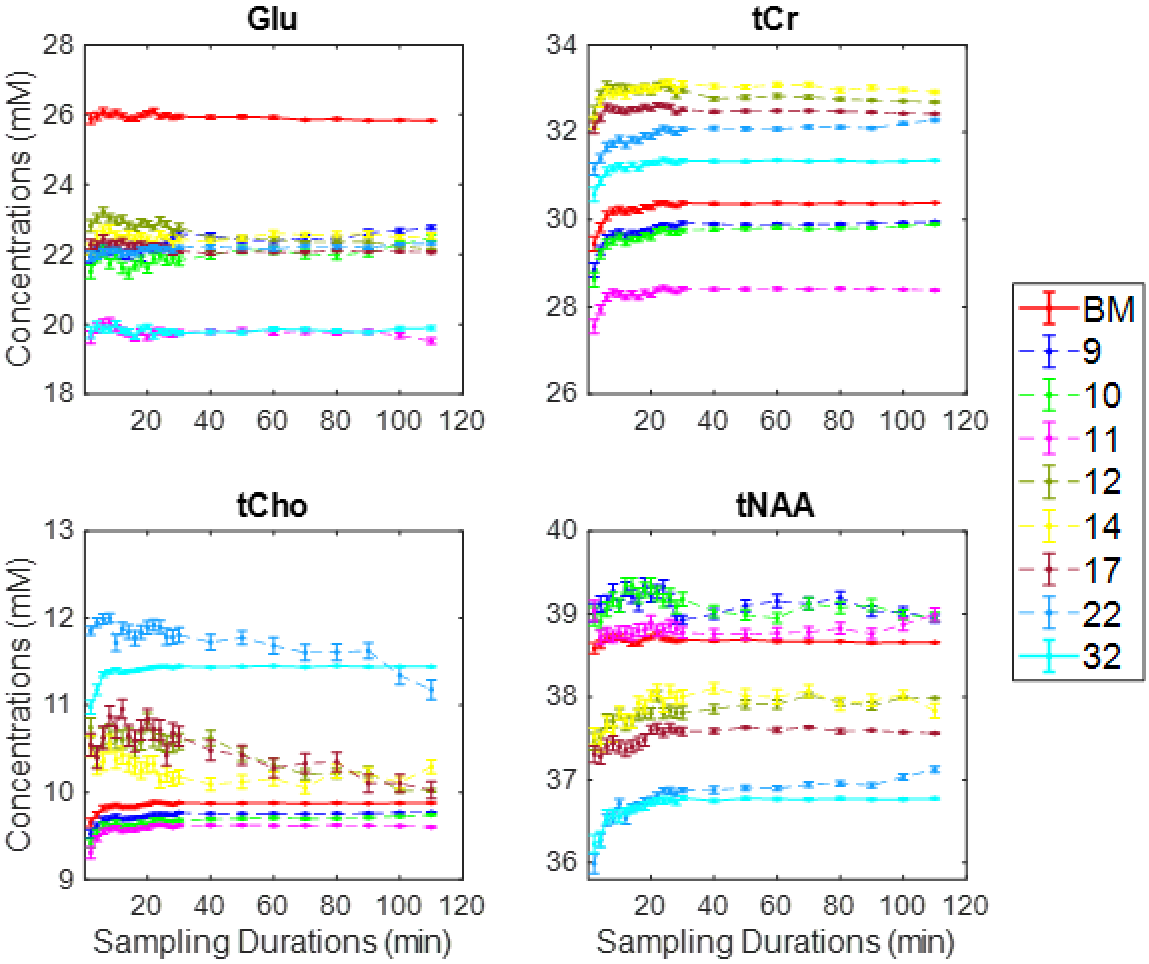


**Figure S2.** Phantom $T_1$ and $T_2$ corrected concentrations with standard error bars for Glu, tCr, tCho and tNAA quantified with 9 optimal basis sets over 20 durations (see **Table 1** for metabolite components in each model). All metabolite concentration estimates show variability between 9 fitted models. BM = base model.

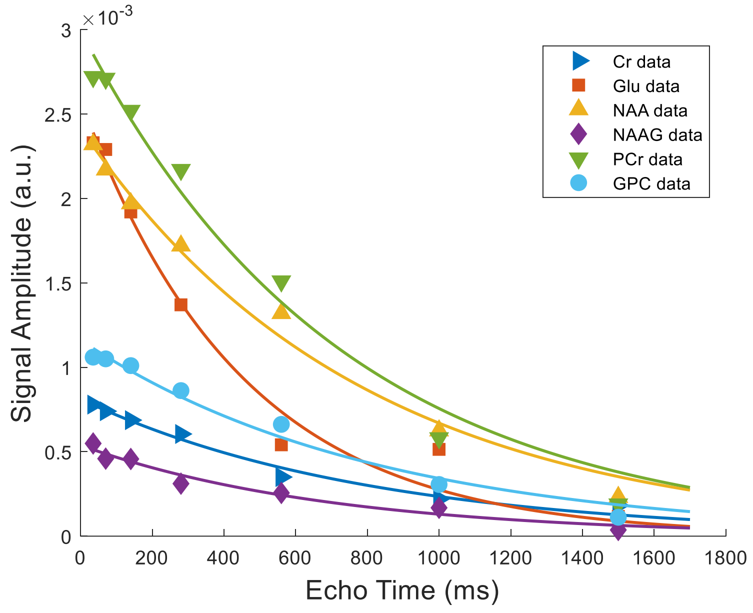


**Figure S3.** Signal vs TE data and fitted curves from which $T_2$ estimates were extracted for Cr, PCr, NAA, NAAG, Glu, and GPC in brain-mimicking phantom acquired at 3 T.

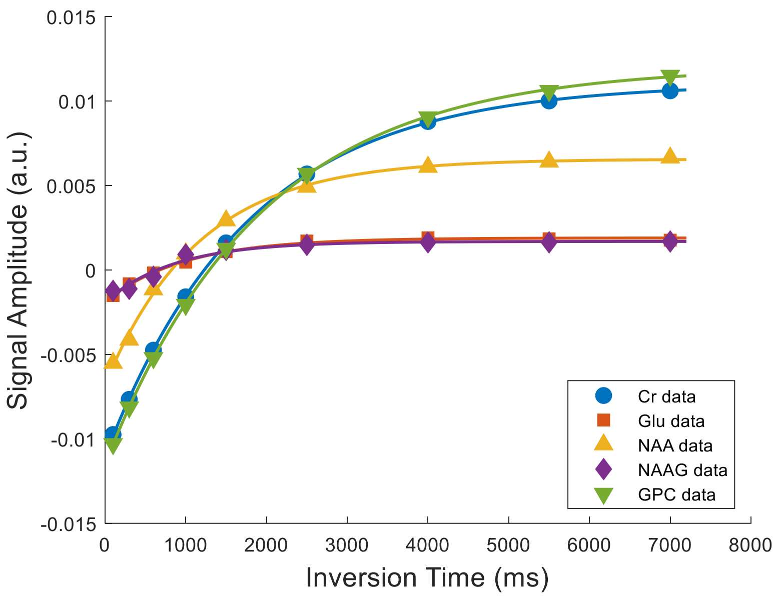


**Figure S4.** Signal vs TI data and fitted curves from which $T_1$ measurements were extracted for Cr, NAA, NAAG, Glu, and GPC in brain-mimicking phantom acquired at 3 T.

## Appendix A3. Optimal basis set model selection.

We use stepwise iterative approach to test models with 7 – 11 components first to inform which additional components improved accuracy and precision, guiding their inclusion in the next more complex models. Base model with 6 prominent signals (Glu, Gln, Cr, GPC, m-Ins, NAA) was always present in the fitting. The results for more relevant models are shown in **Figures S5 – S9**.

Fitting outcomes using a **7-componets model** demonstrated that Glu quantification with GABA, NAAG or GSH components in the model demonstrated lower bias (36 % - 42 % vs > 43 %) and RMSE (4.3 – 4.9 vs > 5) compared to the models with the remaining component combinations. Inclusion of GSH to the base model yielded lower bias for tCr and tNAA signals and inclusion of Glc reduced tCho bias by more than 10 % compared to the base model (18 % vs 33 %). Inclusion of partner metabolites (PCr, NAAG and PCh) for tCr, tNAA and tCho in the basis sets notably increased bias, variability, RMSE and CRLB for all three metabolites (see **Figure S5**).

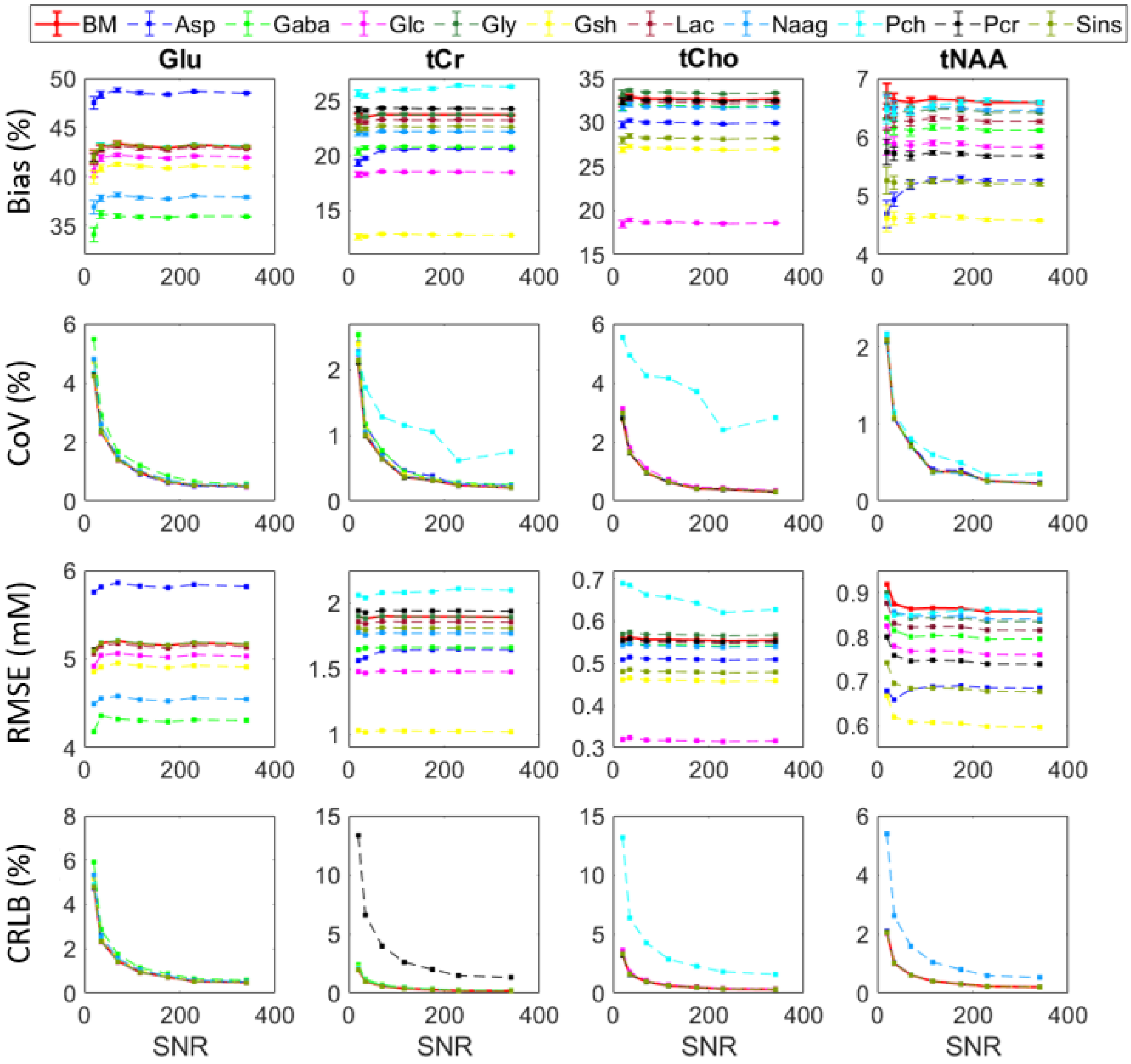


**Figure S5**. Glu, tCr, tCho, tNAA quantification with ten 7-component model variations. Top panel is showing bias from the ground truth (GT, %) with standard error bars, middle panel is showing variability in the data expressed as coefficient of variation (CoV, %), the third panel is showing root-mean-squared error (RMSE, mM) and the bottom panel is showing Cramér-Rao lower bound (CRLB, %) for metabolite estimates uncertainty. BM = base model with 6 components is shown in solid red line. Glu = glutamate, tCr = total creatine, tCho = total choline, tNAA = total N-asetylasparate. BM = base model, Asp = aspartate, Gaba = γ-aminobutyric acid, Glc = glucose, Gly = glycine, GSH = glutathione, Lac = lactate, NAAG = N-asetylaspartylglutamate, PCh = phosphorylcholine, PCr = phosphocreatine, s-Ins = scyllo-inositol. See **Table 1** for the full breakdown of basis sets components.

**The 7-component** model results informed our next comparison, where we assess **8-component models**. Since GABA yielded lower bias for Glu, this component was included in all fittings. Glu quantification with GABA, and Glc, NAAG or GSH components in the base model demonstrated lower bias (23 - 31 % vs > 35 %) and RMSE (2.8 – 3.7 vs > 4) out of all **8-component model** comparisons. There was no reduction in bias for tCr, tCho and tNAA signals when their partner metabolites (PCr, PCh, NAAG) were added to the models. As expected, estimates variability increased with an increased noise level for all metabolites of interest. This was particularly evident for tCho when PCh component was added to the base model (see **Figure S6**).

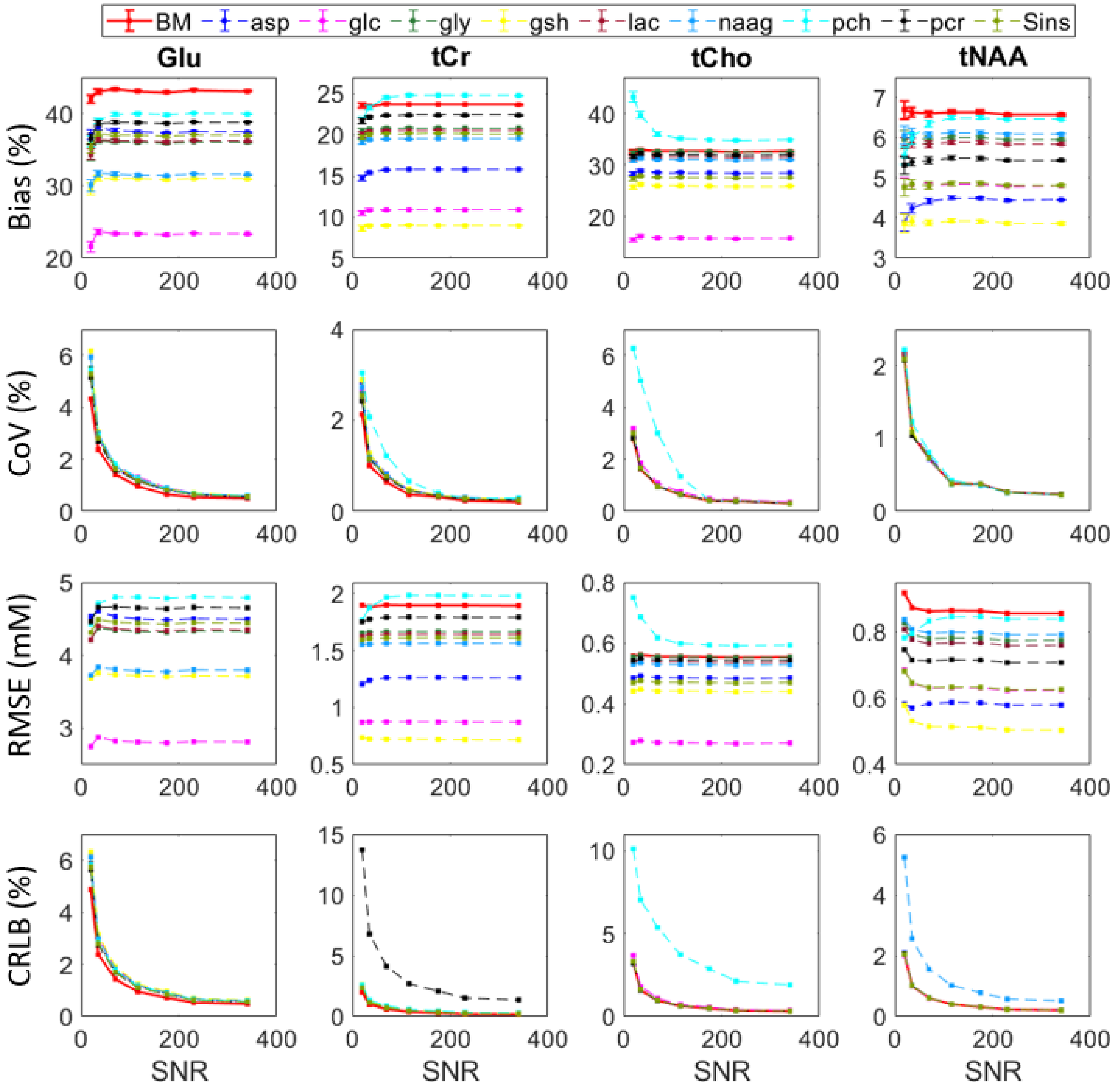


**Figure S6**. Glu, tCr, tCho, tNAA quantification with nine 8-component model variations. GABA component is present in all fittings. BM = base model with 6 components is shown in solid red line.

Following from this, we assessed **9-component models** (see **Figure S7**). Since inclusion of GSH component yielded lower bias for all 4 metabolites of interest, and NAAG component demonstrated lower bias for Glu, both of these components were included in all fittings. The combination of "NAAG GSH" with GABA, Lac, PCr or PCh added to the base model yielded lower bias (20 – 29 % vs > 30 % - 48 %) and RMSE (2.4 – 3.4 vs > 4) for Glu out of all basis signal variations. Base model + "GABA NAAG GSH " showed bias of approximately 20 % for Glu, 24 % for tCho, 6 % for tCr, and 4.5 % for tNAA. Inclusion of partner metabolites for tCr, tCho and tNAA yielded similar results to the **8-component models**.

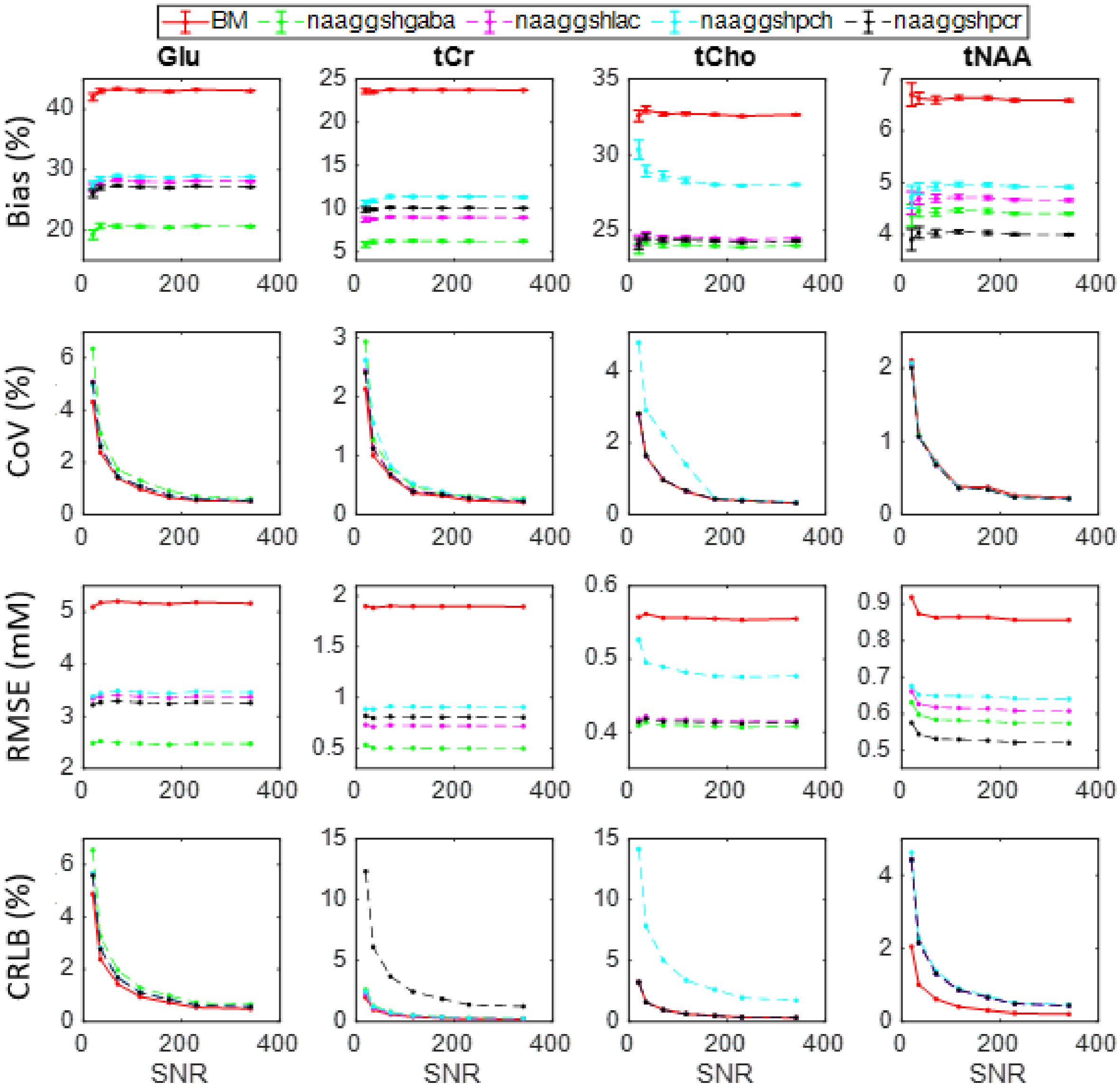


**Figure S7**. Glu, tCr, tCho, tNAA quantification with four 9-component model variations. NAAG and GSH components are present in all fittings with GABA, Lac, PCh and PCr respectively. BM = base model with 6 components is shown in solid red line.

Both fittings with **8-** and **9-component models** informed our next comparison, where we assess **10-component models**. The combination of “NAAG GSH” with GABA or Lac and Asp, Glc, Gly, PCh, PCr, or s-Ins yielded a lower bias (~11 % - 27 % vs > 28 %) and RMSE (1.4 vs > 3.5) for Glu out of all component variations. Inclusion of GABA in the model notably improved fits compared to models where GABA was not present. Base model + “GABA NAAG GSH Glc” showed bias of approximately ~ 11 % for Glu, 3 % for tCr, and < 15 % for tCho. tNAA estimated parameters remained reasonably stable across all comparisons. Inclusion of PCh in the basis models notably increased bias, variability of estimates, RMSE and CRLB for Glu, tCr and tCho (see **Figure S8**).

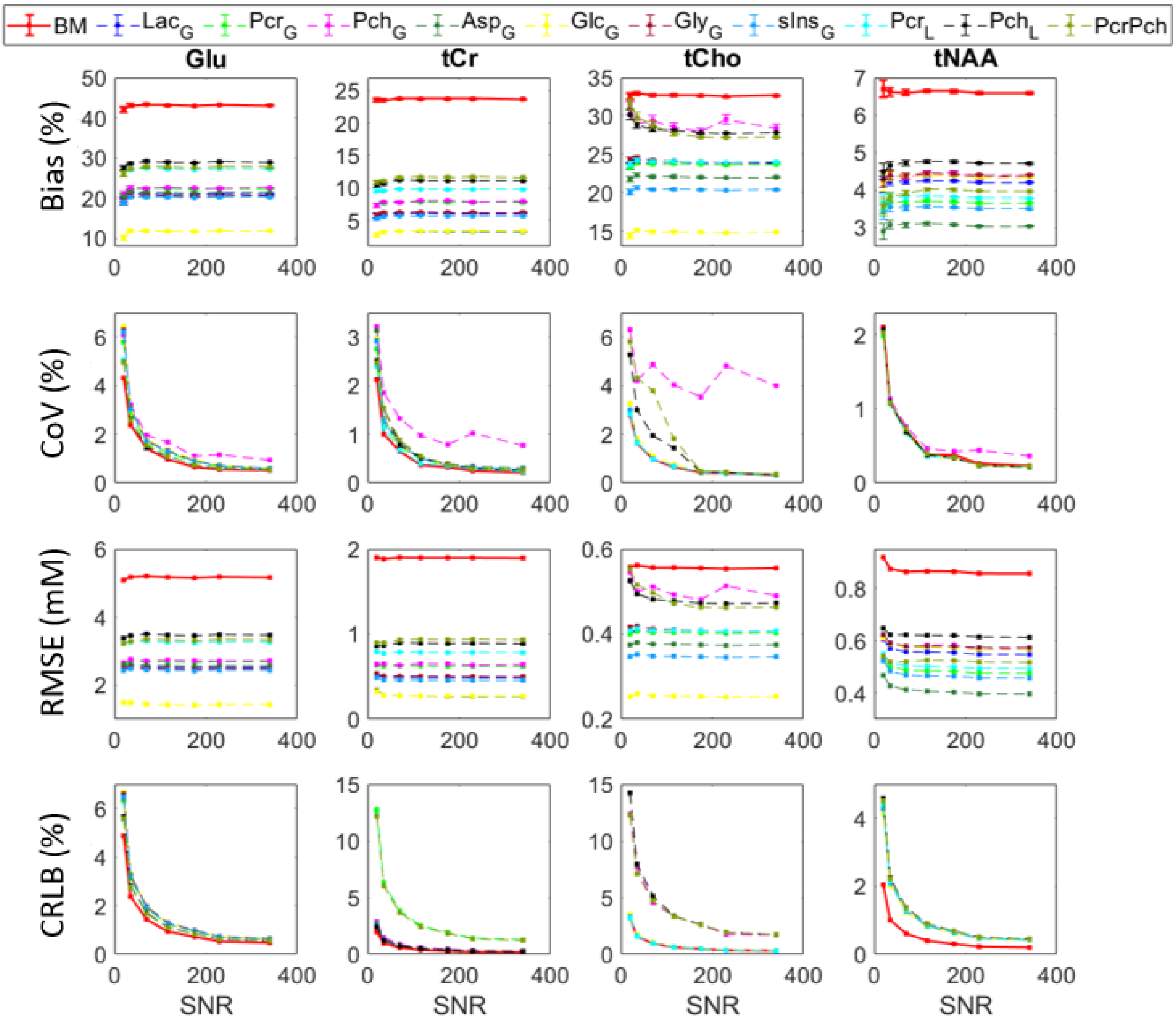


**Figure S8**. Glu, tCr, tCho, tNAA quantification with ten 10-component model variations. NAAG and GSH components are present in all fittings. BM = base model with 6 components is shown in solid red line. “G” = GABA, NAAG, GSH were included in all models. “L” = lactate, NAAG, GSH were included in all models. “PCrPCh” = NAAG, GSH, PCr, PCh were included in all models.

For **11-component models**, since “GABA NAAG GSH” components consistently showed lower bias in 7 – 10 basis component fittings, we include these 3 components in all model fittings. The combination of “GABA NAAG GSH”, with “Lac Glc”, “Lac PCr or PCh”, “PCr PCh” or “Glc Asp or s-Ins” yielded lower bias (12 % - 24 % vs > 30 %) and RMSE (1.5 – 2.8 vs > 4) for Glu out of all metabolite component variations. Models where either GABA, NAAG or GSH components were not included showed greater bias for all 4 metabolites. Bias, variability, RMSE and CRLB parameters for Glu, tCr, tCho demonstrate notable increase when PCh component was added to the models, while tNAA remained fairly stable. CRLB and variability increased with an increased noise level for all metabolites of interest. This was particularly evident for basis sets with PCh component. Base model + “GABA NAAG GSH Glc Lac” showed bias of approximately 12 % for Glu compared to 11 % in **10-component** model (BM + “GABA NAAG GSH Glc”) and 20 % in **9-component** model (BM + “GABA NAAG GSH”). No change was noted in bias for tCr (3.2 % vs 3.3 %) and tNAA (4.2 % vs 4.3 %) between **11-** and **10-component** models, whereas the **9-component model** fitting demonstrated increased bias for tCr (6 %) compared to the **11-component** model fitting (3.3 %). tCho bias is notably reduced (14 % vs 24 %) in the **11-component** model compared to the **9-component** model fitting (see **Figure S9**).

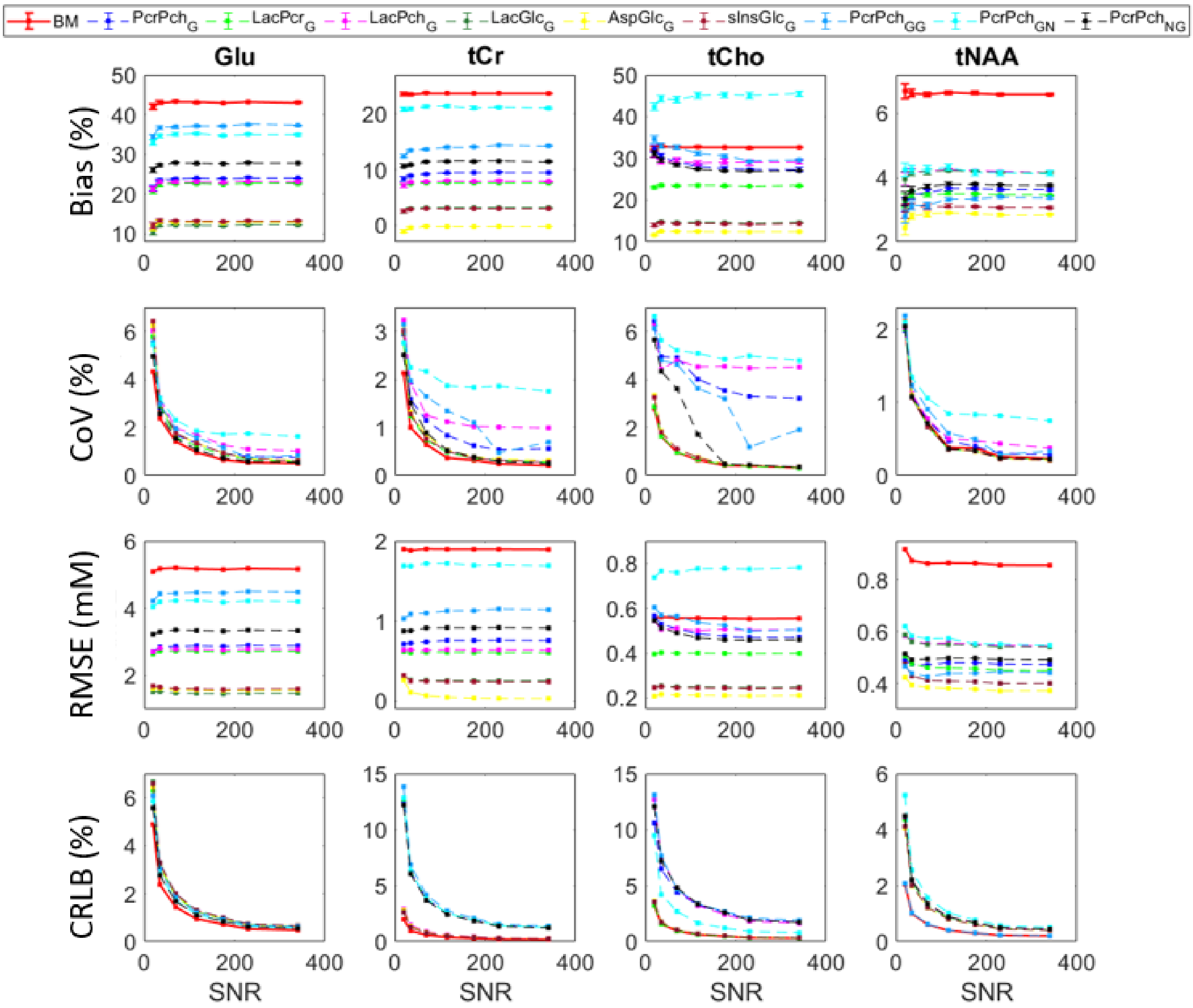


**Figure S9**. Glu, tCr, tCho, tNAA quantification with nine 11-component model variations. BM = base model with 6 components is shown in solid red line. G = “GABA NAAG GSH” present in all models; GG = “GABA GSH LAC” present in the model. GN = “GABA NAAG LAC” present in the model. NG =” NAAG GSH LAC” were included in the model.

## Appendix A4. Background handling selection with QUEST-Subtract.

We used QUEST-Subtract to investigate background handling approaches aimed at improving accuracy and precision in synthetic human brain data analysis. The effects of truncating 15 points (7.5 ms), 20 points (10 ms), 30 points (15 ms), and 40 points (20 ms) on Glu, tCr, tCho, and tNAA signals are shown in **Figures S10 – S13**.

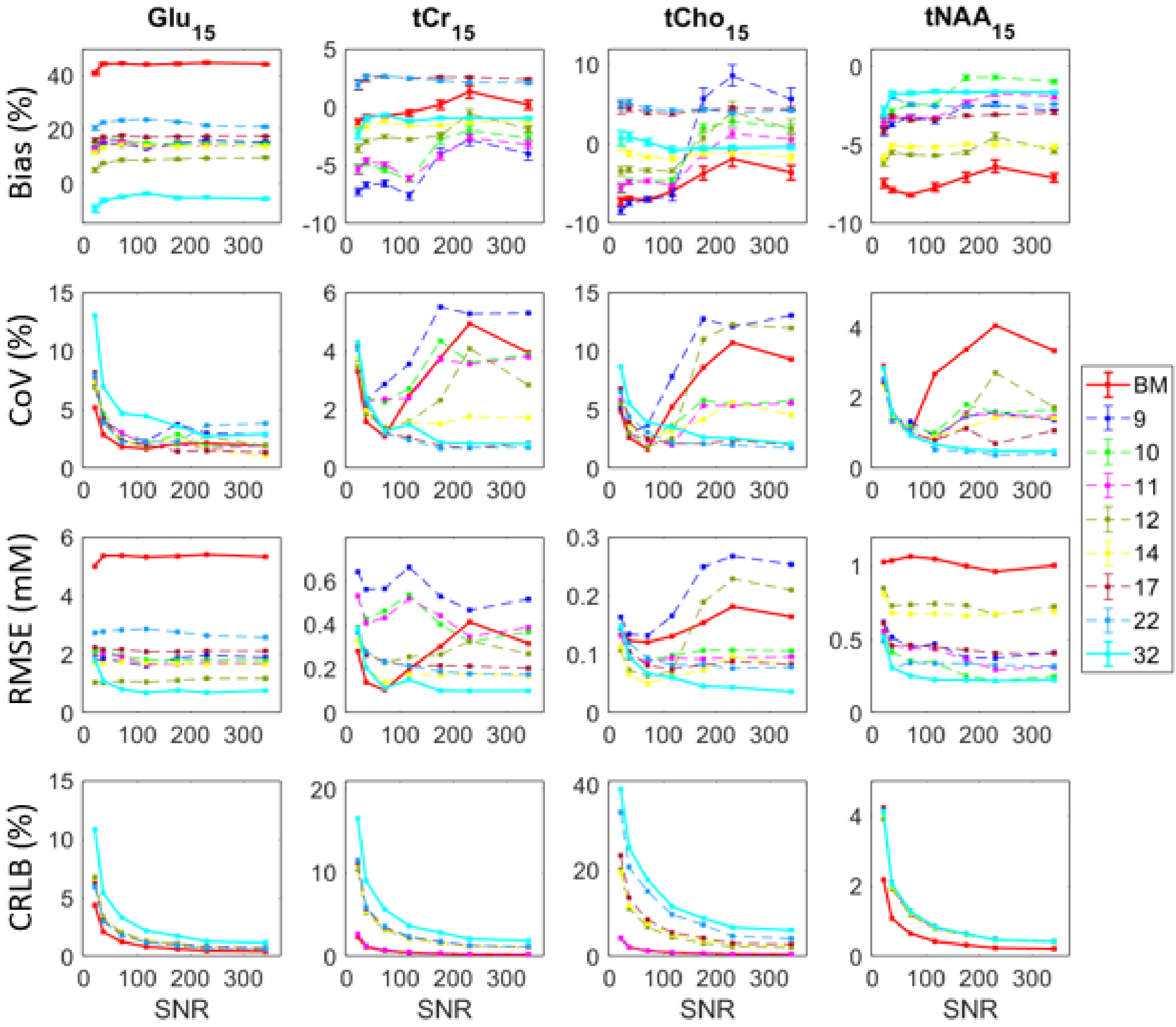


**Figure S10.** Glu, tCr, tCho, tNAA quantification with 9 optimal basis sets across seven SNR levels, using QUEST-subtract background handling with 15 truncated points. Top panel is showing bias from the ground truth (GT, %) with standard error bars, middle panel is showing variability in the data expressed as coefficient of variation (CoV, %), the third panel is showing root-mean-squared error (RMSE, mM) and the bottom panel is showing Cramér-Rao lower bound (CRLB, %) for metabolite estimates uncertainty. BM = base model with 6 components is shown in solid red line. See **Table 1** for the full breakdown of basis sets components.

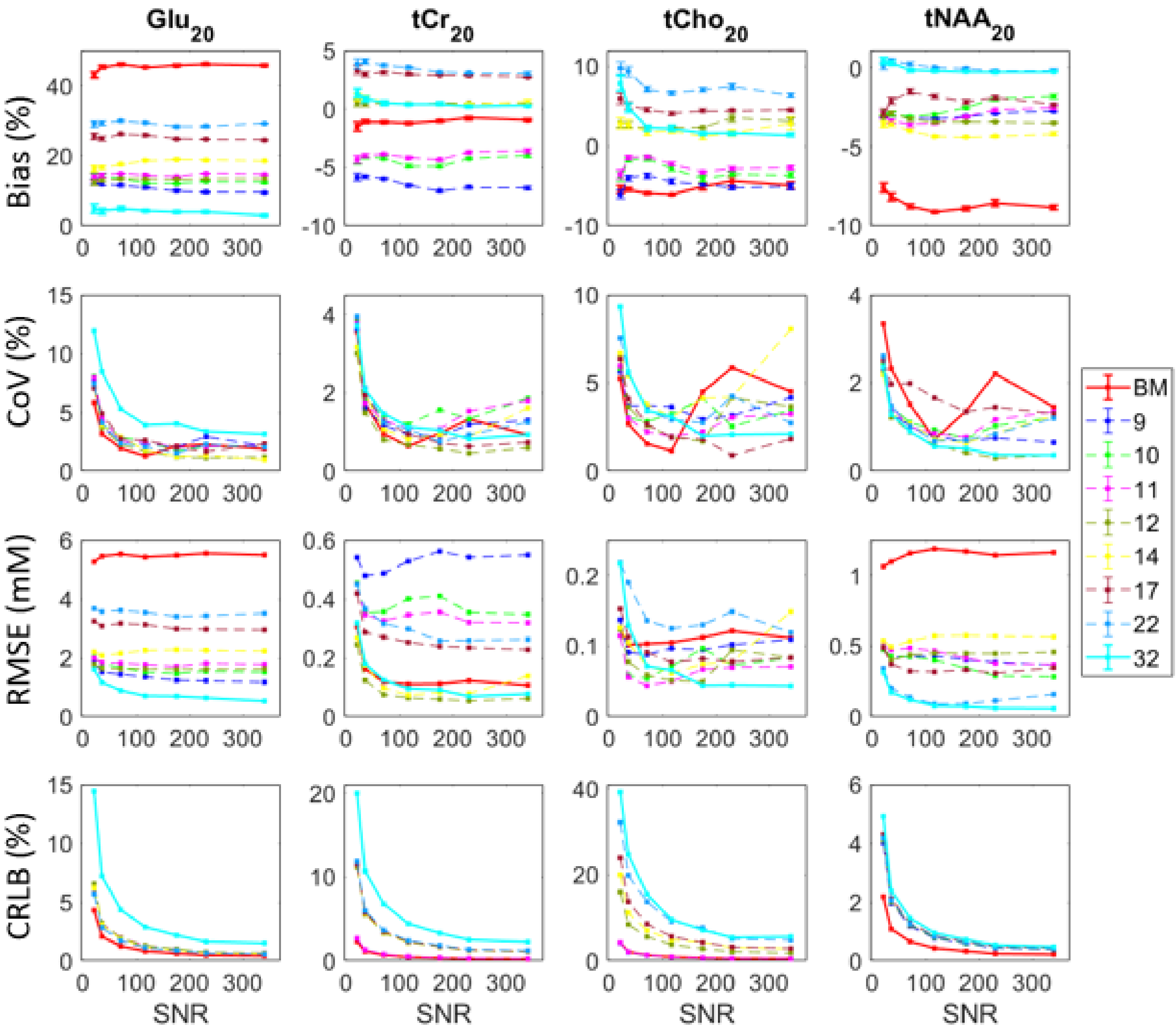


**Figure S11.** Glu, tCr, tCho, tNAA quantification with 9 optimal basis sets across seven SNR levels, using QUEST-subtract background handling with 20 truncated points. BM = base model with 6 components is shown in solid red line. See **Table 1** for the full breakdown of basis sets components.

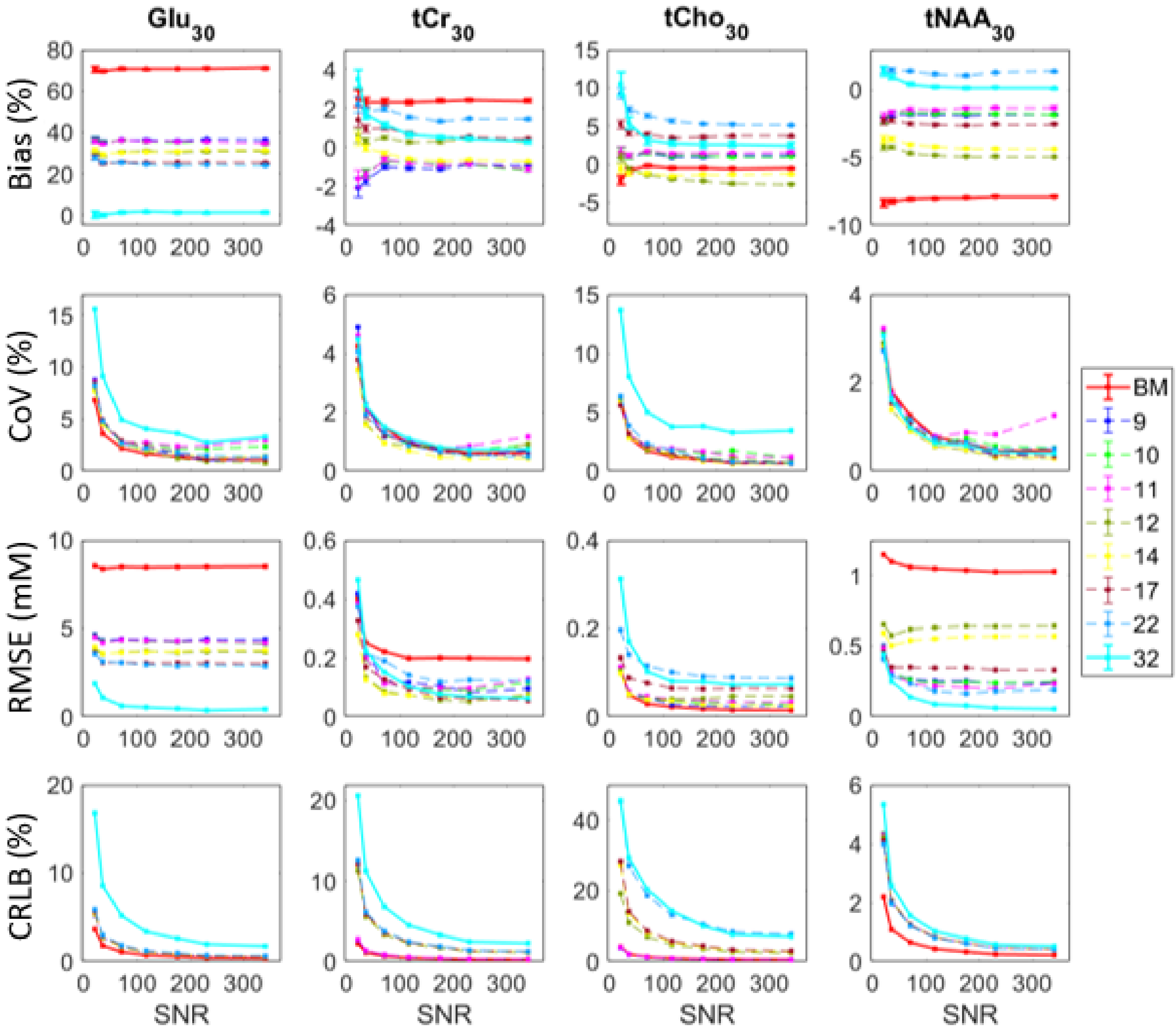


**Figure S12.** Glu, tCr, tCho, tNAA quantification with 9 optimal basis sets across seven SNR levels, using QUEST-subtract background handling with 30 truncated points. BM = base model with 6 components is shown in solid red line. See **Table 1** for the full breakdown of basis sets components.

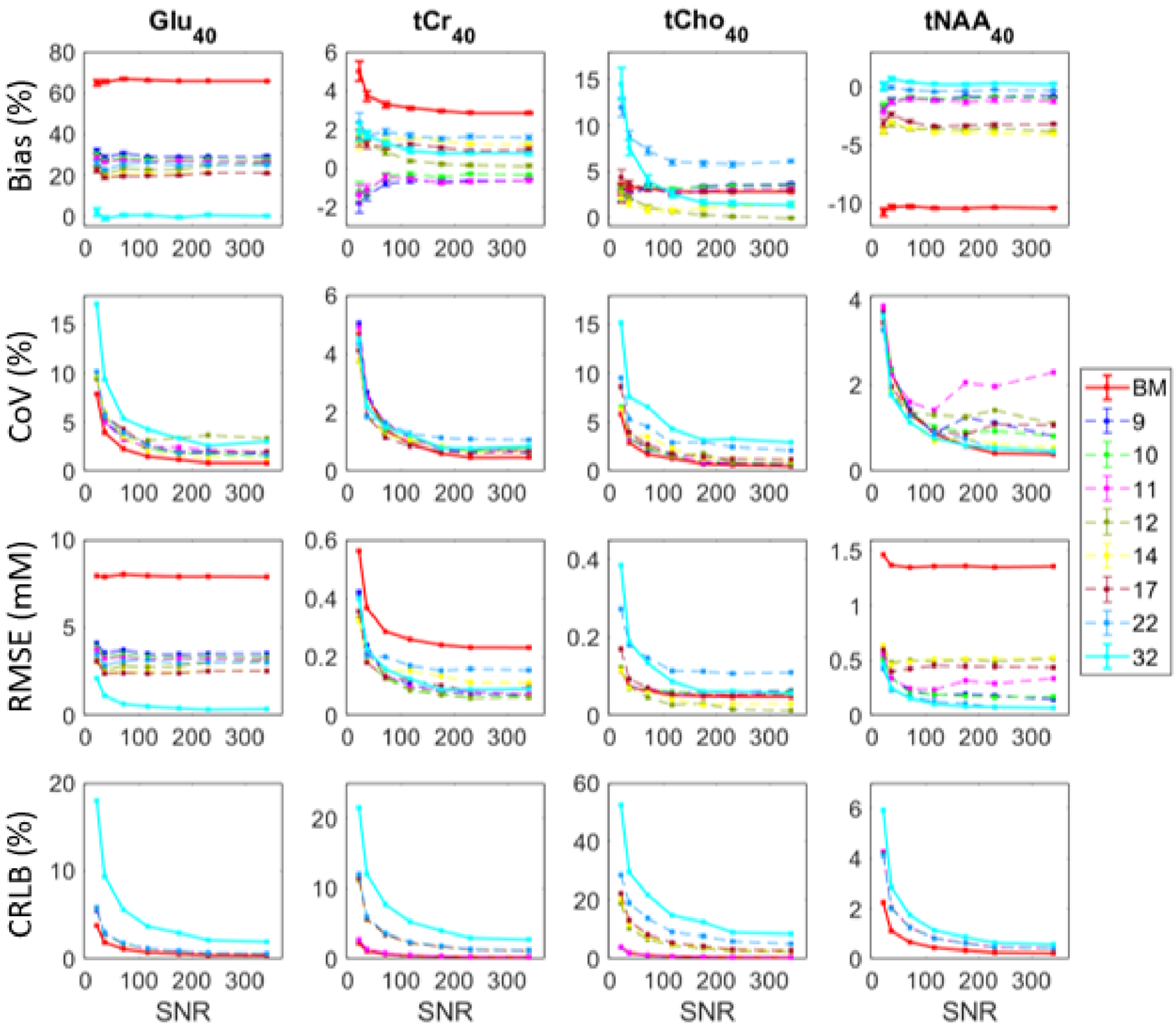


**Figure S13.** Glu, tCr, tCho, tNAA quantification with 9 optimal basis sets across seven SNR levels, using QUEST-subtract background handling with 40 truncated points. BM = base model with 6 components is shown in solid red line. See **Table 1** for the full breakdown of basis sets components.

## Appendix A5. High-resolution spectrometer results.

The AMARES results for high-resolution phantom data acquired with 18.8 T (800 MHz) Bruker spectrometer are shown in the **Table S2.** A difference spectrum between first and last high resolution phantom acquisition is shown in the **Figure S14** below.

**Table S2.** AMARES analysis results of first and last spectrometer acquisition.

| Metabolite | Peak (ppm) | 1st acquisition (mM) | Last acquisition (mM) | Change (%) |
|---|---|---|---|---|
| NAA | 2.02 (s) | 31.9 | 31.9 | 0 |
| Glutamate | 2.355 (m) | 28.7 | 28.6 | 0 |
| Creatine | 3.04 (s) | 24.6 | 24.6 | 0 |
| *Myo*-inositol | 3.28 (t) | 21.1 | 20.5 | -3 |
| Lactate | 1.34 (d) | 12.5 | 12.5 | 0 |
| Glutamine | 2.45 (m) | 7.1 | 7.5 | 5 |
| Phosphocreatine | 3.047 (s) | 11.0 | 11.4 | 4 |
| Choline | 3.205 (s) | 7.2 | 6.9 | -4 |
| Ethanolamine | 3.146 (t) | 7.8 | 7.5 | -3 |
| GABA | 1.904 (m) | 7.0 | 6.9 | -1 |
| NAAG | 2.06 (s) | 5.6 | 5.5 | -3 |
| Taurine | 3.43 (t) | 4.5 | 4.3 | -5 |
| Aspartate | 2.75 (dd) | 5.4 | 5.3 | -2 |
| Glycine | 3.57 (s) | 3.8 | 3.9 | 3 |
| Glycerophosphocholine | 3.232 (s) | 2.6 | 2.5 | -5 |
| Acetate | 1.925 (s) | 2.7 | 2.6 | -2 |
| *Scyllo*-inositol | 3.35 (s) | 1.8 | 1.8 | -1 |

***Note:*** Peak = quantified peak and its multiplicity in ppm, $1^{st}$ and last acquisition quantification results in mM using lactate standard propagated to NAA and Cr respectively. Change in metabolite concentrations after 5 hours of sampling in %.

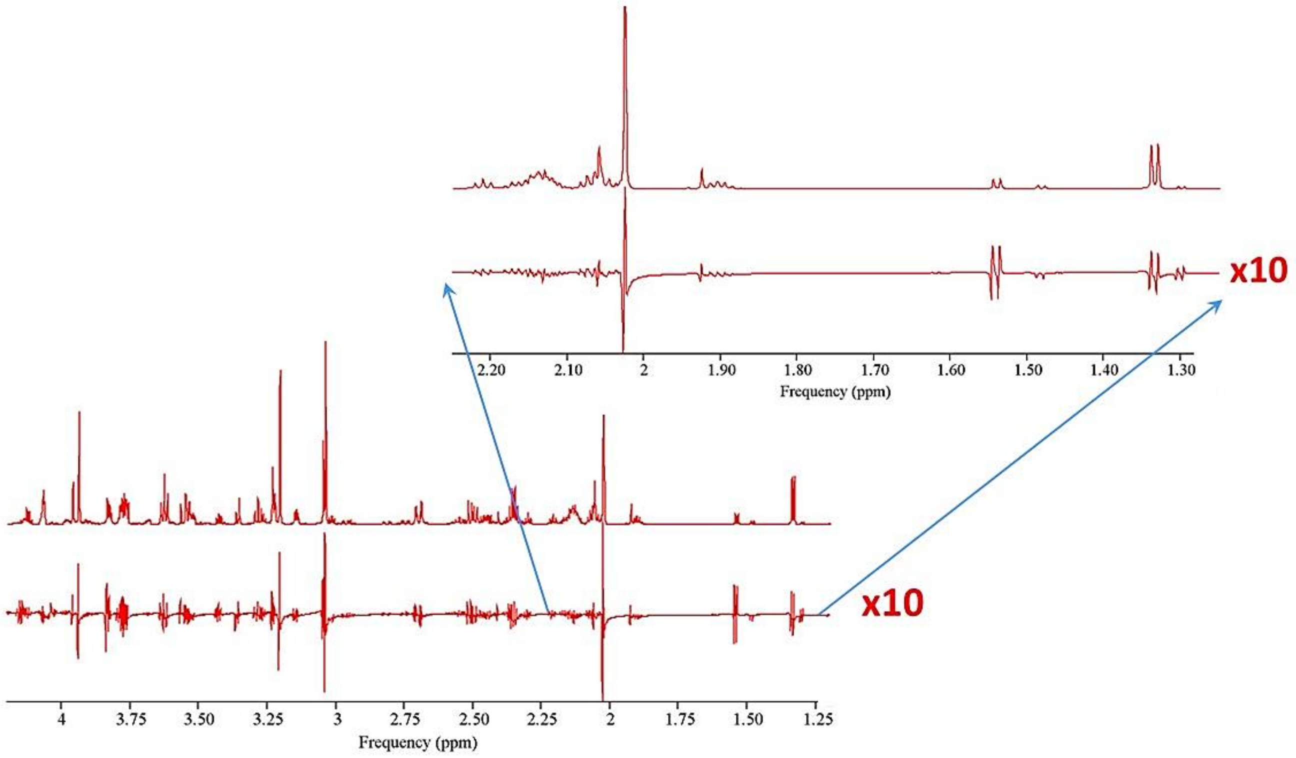


**Figure S14.** Difference spectrum between first and last high resolution phantom acquisition on Bruker spectrometer at 800 MHz.

The lower two traces show the first high resolution (800 MHz) acquisition (top) and the difference spectrum (bottom) between the first and last acquisition (5-hrs later) with the vertical scale multiplied by 10, over the frequency range of 1.2 - 4.2 ppm. The expansion from 1.3 - 2.2 ppm in the upper right shows that there was a small frequency drift across the acquisition time resulting in +ve and –ve difference signals which give the visual appearance of integrating close to zero, indicating negligible concentration changes over 5-hours at room temperature.
