# Supplementary material for: Influence of Basis Set Composition on Metabolite Quantification of 1H‐MRS at 3 T: Combining In Silico, In Vivo and In Vitro Evidence
Source: NMR Biomed. 2026 Feb 11;39(3):e70230. doi: 10.1002/nbm.70230 (PMC12894809; doi:10.1002/nbm.70230)
Supplement: Supplementary file 2 — Appendix S2: MRSinMRS checklist. [file NBM-39-e70230-s002.pdf]

Appendix B: MRSinMRS Checklist.

Here we provide a summary using the minimum reporting standards in MRS. Please see Lin et al. 'Minimum Reporting Standards for in vivo Magnetic Resonance Spectroscopy (MRSinMRS): Experts' consensus recommendations. NMR in Biomedicine. 2021;e4484. doi.org/10.1002/nbm.4448

**Site:** The University of Manchester School of Health Sciences Imaging Research Facility.	
**1. Hardware**	
a. Field strength [T]	3 T
b. Manufacturer	Philips
c. Model (software version if available)	R 5.3
d. RF coils: nuclei (transmit/ receive), number of channels, type, body part	1H, 32 channel, Head
e. Additional hardware	N/A
**2. Acquisition**	
a. Pulse sequence	PRESS
b. Volume of Interest (VOI) locations	ACC, centered along the midline
c. Nominal VOI size [cm^3, mm^3]	30 x 30 x 30 mm^3
d. Repetition Time (TR), Echo Time (TE) [ms, s]	TR = 1500/8000 ms, TE = 35 ms, TE_1/TE_2 = 16/19 ms, TI = 600 ms
e. Total number of Excitations or acquisitions per spectrum<br>In time series for kinetic studies<br>i. Number of Averaged spectra (NA) per time-point<br>ii. Averaging method (e.g. block-wise or moving average)<br>iii. Total number of spectra (acquired / in time-series)	12/5 averages
f. Additional sequence parameters (spectral width in Hz, number of spectral points, frequency offsets)<br>If STEAM: Mixing Time (TM)	2000 Hz, 1024 data points

g. Water Suppression Method	Default, excitation with a window set to 140 Hz
h. Shimming Method, reference peak, and thresholds for “acceptance of shim” chosen	2nd order pencil beam, water
i. Triggering or motion correction method (respiratory, peripheral, cardiac triggering, incl. device used and delays)	No trigger or active motion correction
**3. Data analysis methods and outputs**	
a. Analysis software	jMRUI v7
b. Processing steps deviating from quoted reference or product	None
c. Output measure<br><br>(e.g. absolute concentration, institutional units, ratio) Processing steps deviating from quoted reference or product	Signal Amplitude
d. Quantification references and assumptions, fitting model assumptions	Basis set list: see Table 1 and Figure S1 for all basis model combinations. All simulations were generated in NMRScopeB.<br>In vivo macromolecules spectrum was included in the basis sets for in vivo and BGMM data analysis.<br>Baseline handling: ‘Subtract’ jMRUI with 10 truncated points (5 ms).
**4. Data Quality**	
a. Reported variables<br><br>(SNR, Linewidth (with reference peaks))	SNR, CRLB
b. Data exclusion criteria	Visual inspection
c. Quality measures of postprocessing Model fitting (e.g. CRLB, goodness of fit, SD of residual)	CRLB, SD, bias, RMSE
d. Sample Spectrum	Figure 6
